# Enhancing adoptive CD8 T cell therapy by systemic delivery of tumor associated antigens

**DOI:** 10.1038/s41598-021-99347-0

**Published:** 2021-10-05

**Authors:** Ditte E. Jæhger, Mie L. Hübbe, Martin K. Kræmer, Gael Clergeaud, André V. Olsen, Camilla Stavnsbjerg, Mette N. Wiinholt, Andreas Kjær, Jonas R. Henriksen, Anders E. Hansen, Thomas L. Andresen

**Affiliations:** 1 Department of Health Technology, Technical University of Denmark, 2800 Kongens Lyngby, Denmark; 2 Cluster for Molecular Imaging, Department of Biomedical Sciences, University of Copenhagen, 2200 Copenhagen, Denmark; 3 Department of Clinical Physiology, Nuclear Medicine and PET, University of Copenhagen and Rigshospitalet, 2100 Copenhagen, Denmark; 4 Biotech Research and Innovation Centre (BRIC) and Finsen Laboratory, University of Copenhagen and Rigshospitalet, 2200 Copenhagen, Denmark

**Keywords:** Cancer microenvironment, Tumour immunology, Cancer, Cancer therapy, Cancer immunotherapy, Cancer therapeutic resistance, Drug development

## Abstract

Adoptive T-cell transfer (ACT) offers a curative therapeutic option for subsets of melanoma and hematological cancer patients. To increase response rates and broaden the applicability of ACT, it is necessary to improve the post-infusion performance of the transferred T cells. The design of improved treatment strategies includes transfer of cells with a less differentiated phenotype. Such T cell subsets have high proliferative potential but require stimulatory signals in vivo to differentiate into tumor-reactive effector T cells. Thus, combination strategies are needed to support the therapeutic implementation of less differentiated T cells. Here we show that systemic delivery of tumor-associated antigens (TAAs) facilitates in vivo priming and expansion of previously non-activated T cells and enhance the cytotoxicity of activated T cells. To achieve this in vivo priming, we use flexible delivery vehicles of TAAs and a TLR7/8 agonist. Contrasting subcutaneous delivery systems, these vehicles accumulate TAAs in the spleen, thereby achieving close proximity to both cross-presenting dendritic cells and transferred T cells, resulting in robust T-cell expansion and anti-tumor reactivity. This TAA delivery platform offers a strategy to safely potentiate the post-infusion performance of T cells using low doses of antigen and TLR7/8 agonist, and thereby enhance the effect of ACT.

## Introduction

The immune system’s ability to recognize and eliminate cancer cells has been demonstrated by the remarkable advances of cancer immunotherapy^1^. Central in generating an effective anti-cancer immune response is the mobilization of cancer-reactive T cells. Unfortunately, a substantial number of patients fail to raise or sustain this response^2^. Adoptive cell transfer (ACT) of T cells expanded and modified ex vivo can be used to (re)constitute the tumor-reactive T cell population. Accordingly, ACT has yielded impressive treatment responses, even in patients that are heavily pretreated and/or refractory to first line treatments^3^. Still, some patients, in particular those with solid tumors, fail to respond to ACT. This can have several underlying causes but is partly due to an inability of the transferred T cells to persist and function after infusion^4–6^.

The phenotype of the T cells used for infusion is known to affect the therapeutic efficacy of ACT, with less differentiated T cell subsets being associated with enhanced persistence and improved anti-cancer activity^7,8^. Strategies to sustain a “young” memory phenotype of T cells expanded ex vivo are therefore being explored. These strategies include culturing of T cells with specific cytokine cocktails, small molecule inhibitors^9^ or co-stimulatory molecules^10^ as well as the generation of antigen-specific T cells from induced pluripotent stem cells^11,12^. While these T cells have a high therapeutic potential due to their longevity^7,8,13^, they most likely require post-infusion priming by activated antigen-presenting cells (APCs), such as dendritic cells (DCs), as they will need to differentiate in vivo to acquire a full effector phenotype.

The therapeutic strategy of combining ACT and subcutaneous peptide-based antigen delivery for post-infusion priming has been shown to enhance the effect of differentiated T cells in clinical trials using either DC vaccination^14^ or peptide-based vaccination^15–17^, motivating further development of this therapeutic approach. The conventional vaccine strategy, based on peptide antigen depot formation, may not be the most effective way to support ACT engraftment and expansion. Conventional adjuvants such as Montanide, has been associated with the induction of dysfunctional T cells at the injection site, and could therefore be problematic for combination with ACT^18–21^. Alternatively, drug delivery strategies that enhance lymph node (LN) accumulation after subcutaneous injection have shown promise for the priming of endogenous T cells in preclinical studies. This includes linking peptide antigens to albumin-binding phospholipid polymers^22^, synthetic high-density lipoprotein nanodiscs^23^, nanogels^24^, synthetic polymeric nanoparticles^25^ and other functionalized biomaterials. In line with this, a recently published preclinical study demonstrated that adoptively transferred CAR-T cells can be primed by amphiphile CAR-T ligands^26^.

Like the tumor microenvironment, the tumor-draining lymph node (tdLN) can be dominated by immune-suppressive subsets that counteract productive T cell responses^27^ and might therefore be a suboptimal anatomical location for T cell priming. As an alternative, the spleen is rich in APCs that could be targeted for antigen-delivery, and biodistribution data suggests the spleen to be a relevant organ for expansion of adoptively transferred T cells^28^. Thus, we hypothesized that TAA-delivery targeted to the spleen could be favorable for expansion of adoptively transferred T cells with both a non-activated and differentiated phenotype. In support of this, systemic vaccination with particles carrying mRNA has recently been shown to yield impressive responses to neo-epitope vaccination in preclinical and clinical studies^29,30^ and systemically injected liposomes carrying OVA antigen has been used to prime endogenous anti-tumor CD8^+^ T cells in preclinical studies^31^. We sought to investigate a therapeutic strategy for the priming of CD8^+^ T cells that are therapeutically inactive unless they are primed following infusion. To achieve this, we explored the concept of targeting TAAs to the spleen, which is as a main organ for nanoparticle accumulation. We found that systemically injected, and not subcutaneously injected, nanoparticles follow the bio-distribution pattern of adoptive transferred T cells, allowing for stimulation of CD8^+^ T cells by establishing immunological synapses with cross-presenting DCs. The nanoparticle-based TAA-delivery platform safely induced priming and expansion of unstimulated adoptively transferred antigen-specific CD8^+^ T cells in the spleen at very low antigen and TLR7/8 agonist doses and in the absence of prior lymphodepletion. We showed that this was followed by extensive tumor infiltration, which significantly improved tumor growth control. Conceptually, we present a novel way of safely achieving T cell engraftment and anti-tumor reactivity to improve responses of T cell treatments, either with naturally occurring or TCR engineered cells. This can potentially be used in new treatment concepts for patients receiving ACT as the field matures and better control of inhibiting T cell differentiation during ex vivo expansion is found.

## Results

### Establishing a versatile antigen delivery platform suitable for systemic administration

**Figure 1:**
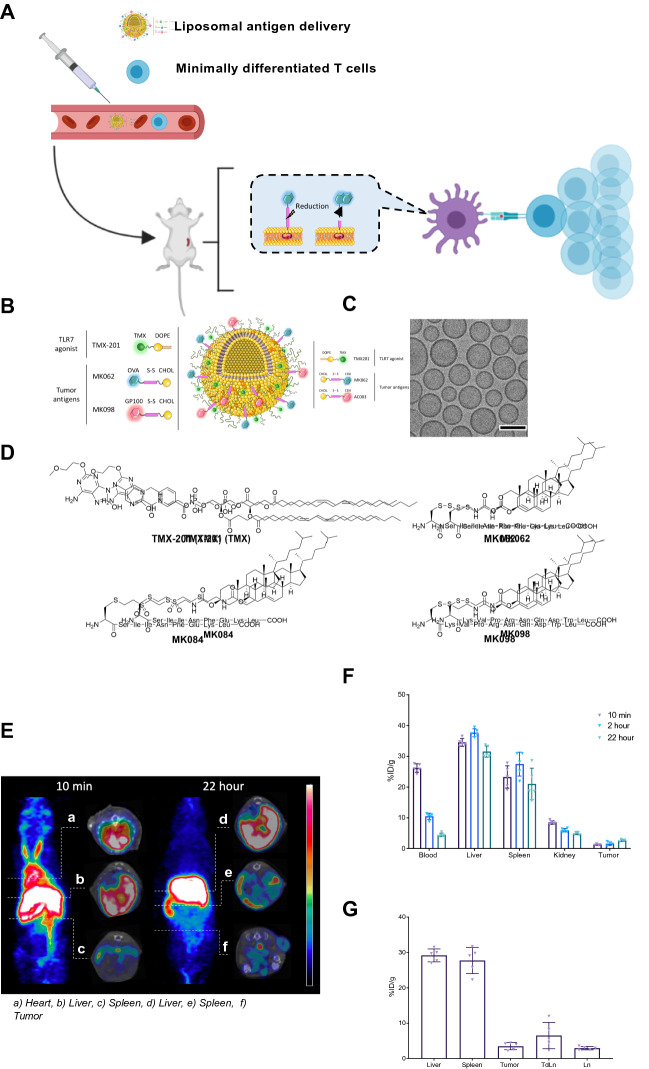
Therapeutic strategy, liposomal antigen delivery vehicle design and characterization. (**A**) Proposed strategy for in vivo priming of minimally differentiated antigen-specific T cells using the liposomal delivery platform. Antigen-specific T cells are infused intravenously (IV) to tumor-bearing recipient mice. 24 h after T cell infusion, mice receive an IV injection with liposomal antigens. The liposomes are designed for internalization by antigen presenting cells, after which intracellular release of peptide antigen occurs due to the redox sensitive linker design. This allows peptide loading to MHC molecules and peptide presentation and priming of antigen-TCR matched T cells. (**B**) Illustration of the liposomal antigen delivery system. (**C**) Cryogenic transmission electron microscopy imaging of liposomal formulation MK098:TMX. Scale bars depict 100 nm. (**D**) Structure of TLR7/8 agonist TMX-201 and peptide antigens coupled to cholesterol via reducible (MK062 and MK098) or non-reducible (MK084) linkers. (**E**) Maximum intensity projection (MIP) of PET scans performed 10 min (left) and 22 h (right) after the injection of radiolabeled liposomes (scale 0–30%ID/g). PET/CT inserts demonstrate 10 min PET activity in the heart ventricles (top), liver (middle) and spleen (bottom) and 22 h PET activity in the liver (top), spleen (middle) and tumor (bottom) (scale liver, spleen, and heart 0–20%ID/g, tumor 0–15%ID/g). (**F**) PET liposome activity and (**G**) post mortem gamma counted ^64^Cu-liposome activity 48 h after injection (mean ± SEM %ID/g).

With the emergence of protocols that focus on the use of minimally differentiated T cells for ACT, we wanted to explore the concept of priming T cells in the host by intravenous (IV) TAA delivery. Naïve or minimally differentiated T cells traffic to lymphoid organs via expression or surface molecules such as L-selectin (CD62L)^32^. Correspondingly, IV*-*injected, nanocarrier systems such as liposomes have been shown to accumulate in the spleen ^31,33^. The biodistribution of liposomal delivery systems can therefore potentially deliver a concise, potent stimulation of T cells in the spleen (Fig. 1A), which cannot be achieved using unformulated peptides and adjuvants.

To achieve this, a liposomal platform system was designed based on co-formulation of a potent TLR7/8 agonist and tumor antigens incorporated into the surface through flexible reducible linkers that upon intracellular reduction release the antigen within phagocytic cells (Fig. 1A). A lipidated TLR7/8 agonist (TMX-201) and cholesterol-conjugated antigens were anchored in the liposome membrane (Fig. 1B), forming homogeneous unilamellar liposomes as confirmed by cryoTEM imaging (Fig. 1C).

Antigens OVA_257–264_ (SIINFEKL) and human gp100_25−33_ (KVPRNQDWL) were synthesized with N-terminal cysteine modifications to facilitate conjugation to cholesterol with or without reducible linkers (Fig. 1D, supplementary materials S1). The physicochemical characteristics of the different formulations, including size, polydispersity (PDI), ζ -potential (Z-pot) and concentrations of lipid, TLR7/8 adjuvant (TMX-201) and antigens are presented in Supplementary Fig. S2. The liposome delivery system formulated with TMX-201 and SIINFEKL antigen conjugated with the reducible linker technology had a particle size of 114.0 nm, PDI of 0.1 and Z-pot of − 15.0 mV, and will hereafter be referred to as MK062:TMX. The non-cleavable liposomal formulation containing the TMX-201 and the SIINFEKL antigen conjugated using a non-reducible linker was 112.0 nm in size, PDI of 0.1 and Z-pot of − 19.8 mV, and is referred to as MK084:TMX. The liposome formulation composed of TMX-201 and the cleavable antigen KVPRNQDWL depicted a size of 123.6 nm, PDI of 0.1 and Z-pot of − 17.6 mV, is denoted as MK098:TMX.

To characterize the biodistribution of the liposomes PET/CT imaging was performed using radiolabeled (^64^Cu-DOTA) MK062:TMX liposomes. PET scans were performed at 10 min, 2 h and 22 h after injection (Fig. 1E). After 48 h, organs were harvested for gamma counting. The PET scans confirmed that the liposomes accumulated in the spleen having a mean spleen activity of 21(± 5) %injected dose/gram tissue (%ID/g) at the 2-h scan (Fig. 1F), and an equivalent activity was also recorded by gamma counting 48 h after injection (Fig. 1G).

### Liposomal antigen delivery renders minimally differentiated T cells therapeutically active

**Figure 2:**
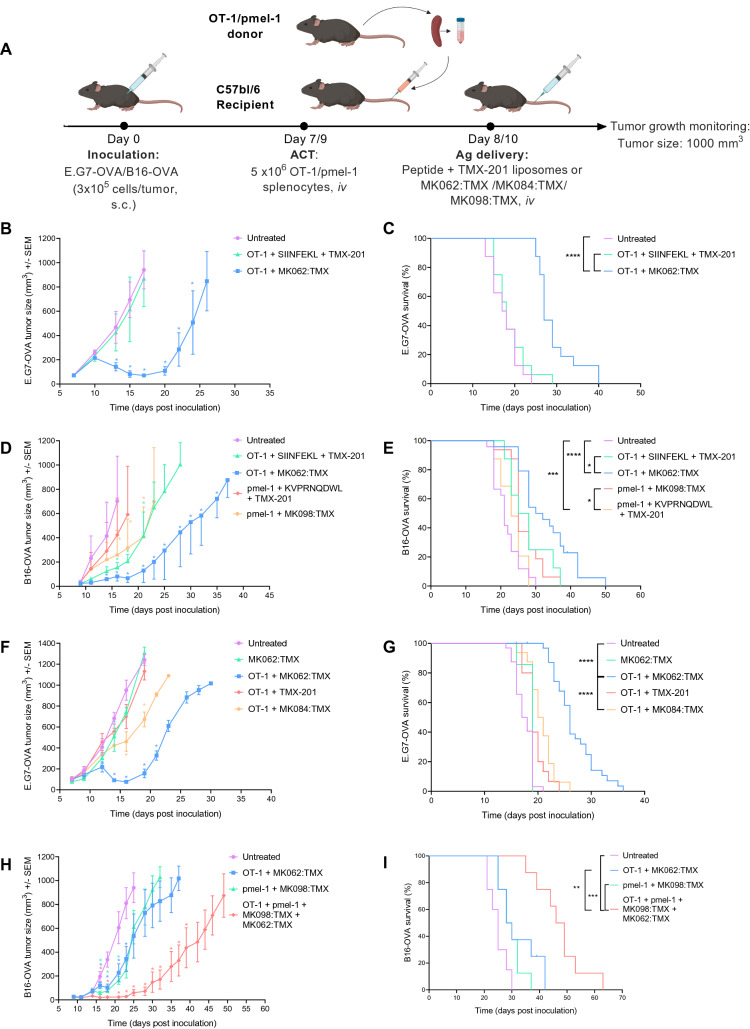
Combining minimally differentiated T cells and systemic antigen delivery improves tumor control and prolongs survival in two syngeneic mouse cancer models. (**A**) Schematic illustration of the treatment schedules applied. Tumor growth curves (**B,D**) and survival plots (**C,E**) of mice receiving either soluble peptide (SIINFEKL or KVPRNQDWL) and liposomal TLR7/8 stimulation (TMX-201) or liposome antigen delivery (MK062:TMX or MK098:TMX) post T cell infusion. Tumor growth curves (**F**) and survival plots (**G**) of mice receiving different liposome formulations post T cell infusion. Tumor growth curves (**H**) and survival plots (**I**) of mice receiving dose-matched combination therapy directed against a single antigen or both antigens. Graphs represent pooled averages from three independent experiments with 8 mice pr. group in each experiment. Graphs (**B**,**D**,**F**,**H**) illustrate mean ± SEM. Survival in (**C**,**D**,**E**,**G**,**I**) was calculated by Log-Rank. Difference in mean tumor size on individual days in (**B**,**D**,**F**,**H**) was calculated using linear mixed model. *P ≤ 0.05, **P ≤ 0.01 ***P ≤ 0.001 ****P ≤ 0.0001.

The therapeutic effect of T cells and systemic liposome mediated antigen delivery was evaluated in syngeneic murine cancer models. OT-1 splenocytes were isolated from TCR-transgenic OT-1 mice (C57BL/6-Tg(TcraTcrb)100Mjb/J) and primed 24 h following infusion with the cognate antigen OVA_257–264_ (SIINFEKL). The antigen was delivered either as peptide dissolved in PBS together with TMX-201 liposomes, or formulated with TMX-201 in liposomes using the reducible linker system (MK062:TMX). A similar set-up was used to prime pmel-1 splenocytes (B6.Cg-Thy1a/Cy Tg(TcraTcrb)8Rest/J) with the cognate antigen gp100_25–33_ (KVPRNQDWL) as soluble peptide or inserted with a reducible linker in a liposomal formulation (MK098:TMX) (Fig. 2A).

Initially, the co-formulation (MK062:TMX) was compared to delivery of the soluble peptide antigen and TMX-201 (TLR7/8 agonist) liposomes as separate components. The soluble peptide and TMX-201 liposomes displayed no or minimal therapeutic effect in combination with transferred T cells. Encouragingly, the combination of ACT and MK062:TMX liposomes induced marked regression of established tumors and significantly prolonged survival in the syngeneic murine E.G7-OVA lymphoma (Fig. 2B,C) and B16-OVA melanoma cancer model (Fig. 2D,E).

ACT therapies come with the risk of cytokine-related toxicities, which can be detrimental to the patients^34^. In these studies, we managed to achieve the therapeutic effect using a single injection of liposomes carrying very small amounts of antigen and adjuvant (down to 10 ng/dose). In addition, we did not observe therapy-induced weight loss exceeding 3% of the initial bodyweight, even for doses 100× of the therapeutically efficient dose (Supplementary Fig. S3).

Neither ACT nor MK062:TMX liposomes had significant therapeutic effect as monotherapies, reflecting that young phenotype and non-activated T cells do not obtain sufficient antigen stimulation in a tumor-setting and a low dose of antigen cannot alone raise a sufficient response (Supplementary Fig. S4). Even though the concept we are investigating in the present work was to support the development of minimally differentiated T cells because of their greater therapeutic potential, we also tested the therapeutic efficacy of combining the liposomal platform with ACT of activated ex vivo expanded T cells, which predominantly consisted of CD8+ T cells with T_CM_ and T_EM_ phenotypes (Fig. S5C). As observed for the minimally differentiated T cells, the liposomal TAA-delivery also improved the efficacy of these cells, pointing towards a potential broader application of this platform for post-infusion priming (Supplementary Fig. S5). Next, we investigated the therapeutic significance of conjugating the antigen by the reducible linker system in MK062:TMX since a reducible linker has previously been shown to enhance cross-presentation for intradermally injected particles^35^. To address this, SIINFEKL was conjugated to cholesterol for liposome insertion using a non-reducible (MK084:TMX) and TMX-201 liposomes without antigen as controls. TMX-201 had no anti-tumor effect when combined with ACT, whereas MK084:TMX resulted in a slight tumor growth delay in combination with ACT (Fig. 2F). The anti-tumor effect of MK084:TMX did however not translate into an improved survival, demonstrating that the presence of the active release mechanism in the liposome system is critical for the therapeutic activity (Fig. 2G).

The design behind the flexible antigen delivery system is based on the idea of activating multiple adoptively transferred T cell clones simultaneously and thereby avoid tumor escape caused by increased selection pressure and accelerated antigen loss^36–39^. Using the B16-OVA model, others have shown that co-transfer expanded OT-1 and pmel-1 T cells can prolong the therapeutic efficacy of ACT^40^. To address if our TAA-delivery platform could be used to expand two different T cell clones simultaneously, we combined OT-1 and pmel-1 splenocyte transfer with antigen delivery of two antigen epitopes for OVA and gp-100 (MK062:TMX and MK098:TMX, respectively). This dual-targeting approach resulted in more sustainable tumor regression and significantly prolonged survival in the B16-OVA model compared to a similar absolute cell dose of either mono-targeting therapy (Fig. 2H,I).

Chemotherapy regimens resulting in lymphodepletion and systemic cytokine release are used as standard preconditioning of patients receiving ACT to enhance the therapeutic effect of the transferred T cells. This combination treatment has resulted in substantial toxicity and even deaths in clinical trials^3^, but is nevertheless continued in the clinic to ensure engraftment of T cells. To address if efficacy of our treatments was further improved by preconditioning, we tested a standard regimen of 200 mg/kg cyclophosphamide (CPX) administered 1 day prior to ACT. Apart from a small delay in tumor growth caused by the chemotherapy, CPX preconditioning did not significantly alter the therapeutic outcome, suggesting that maximal therapeutic efficacy could be achieved in the absence of lymphodepleting chemotherapy using the liposome antigen delivery system (Supplementary Fig S6).

### Intravenous liposomal antigen delivery activates and induce antigen presentation by splenic DCs

**Figure 3:**
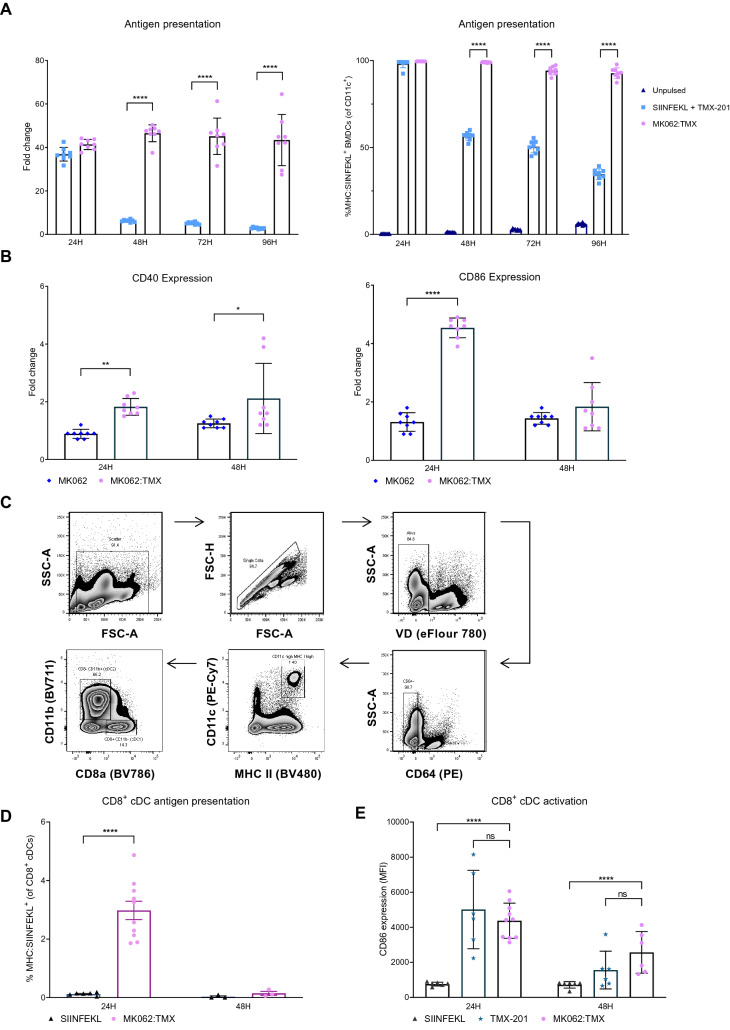
MK062:TMX liposomes effectively activate and deliver antigen to APCs in vitro and in vivo*.* (**A**) Evaluation of SIINFEKL antigen presentation on CD11c^+^ BMDCs 24–96 h after treatment with MK062:TMX liposomes or soluble SIINFEKL peptide + TMX-201 liposomes depicted as fold change to unpulsed controls (left) and percentage of SIINFEKL antigen-presenting BMDCs (right). (**B**) Expression (MFI) of activation markers CD40 and CD86 on CD11c^+^ BMDCs in response to treatment with MK062:TMX or MK062 (without TMX-201 TLR7/8 agonist) liposomes. (**C**) Antigen presenting CD8α^+^ cDCs were identified as viability dye (VD)^-^, CD45^+^, CD64^-^, CD11c^high^, MHC II^high^, CD11b^-^, CD8α^+^ and MHC:SIINFEKL^+^. (**D**) Antigen presentation by splenic CD8α^+^ cDCs after treatment with MK062:TMX or a corresponding dose of soluble SIINFEKL peptide, depicted as MHC:SIINFEKL^+^ CD8α^+^ cDCs (**E**) Expression of activation marker CD86 on splenic CD8α^+^ cDCs in response to MK062:TMX, SIINFEKL or TMX-201 treatment. Graphs represent pooled data from 2–4 independent experiments with n = 2–6 for each experiment. Error bars represent the mean ± SD. Differences were calculated with students T-test for A and B and ordinary one-way ANOVA for C and D *P ≤ 0.05, **P ≤ 0.01 ***P ≤ 0.001 ****P ≤ 0.0001.

Successful priming of tumor-reactive T cells is critically dependent on tumor antigen uptake, processing and presentation on MHC molecules by APCs. To evaluate the antigen presentation induced by MK062:TMX, we quantified the SIINFEKL presentation on CD11c^+^ bone-marrow derived dendritic cells (BMDCs) by flow cytometry using an antibody specific for SIINFEKL peptide presented on MHC I (H-2Kb) molecules. By assessing the SIINFEKL antigen presentation over four consecutive days, we found that BMDCs treated with MK062:TMX exhibited a higher level of SIINFEKL presentation than BMDCs treated with soluble SIINFEKL antigen + TMX-201 after 48–96 h in culture (Fig. 3A). The MK062:TMX induced antigen presentation persisted at a stable level for 96 h in culture whereas SIINFEKL + TMX-201 induced antigen presentation decreased significantly after 48 h. This extended period of antigen presentation was an encouraging finding, since a sustained antigen-presentation has been associated with enhanced expansion and effector function of antigen-specific T cells in vivo^41–43^.

To confirm that TMX-201 was capable of activating BMDCs, the expression of the activation markers CD86 and CD40 was evaluated at 24 and 48 h after treatment with MK062:TMX liposomes or MK062 liposomes without TMX-201 TLR7/8 agonist. After 24 h, CD11c^+^ BMDCs treated with MK062:TMX liposomes had significantly upregulated expression levels of both CD86 and CD40 compared to MK062 treated BMDCs. After 48 h, the expression of CD86 had returned to baseline whereas CD40 remained upregulated (Fig. 3B).

Next, it was evaluated if MK062:TMX liposomes could induce antigen presentation and activation of APCs in vivo. As the liposomal formulation used for the MK062:TMX delivery platform accumulates in the spleen, the activation and SIINFEKL antigen presentation on MHC I molecules was evaluated on splenic APCs. Conventional dendritic cells type I (cDC1s) are known to excel in cross-presentation of extracellular derived antigen and have been reported as indispensable for mounting an anti-tumor T cell response^44–46^. Since the platform for TAA delivery used in this context necessitate extracellular uptake and cross-presentation by APCs for T cell priming, we evaluated the antigen-presentation by splenic CD8α^+^ cDC1s. For this purpose, female C57bl/6 mice received a single IV injection with MK062:TMX liposomes corresponding to a treatment with 10 µg SIINFEKL epitope. Control mice were treated IV with 10 µg soluble SIINFEKL peptide dissolved in PBS. After 24 h the splenocytes were stained for flow cytometry analysis and CD8α^+^ cDC1s were identified as viability dye (VD)^-^, CD45^+^, CD64^-^, CD11c^hi^, MHC II^hi^, CD11b^-^ and CD8α^+^ (Fig. 3C). The MK062:TMX liposomes induced SIINFEKL antigen presentation levels by splenic CD8α^+^ cDC1s (MHC:SIINFEKL^+^) to a higher extent than observed in mice treated with soluble SIINFEKL antigen (Fig. 3D). SIINFEKL antigen presentation was also evaluated on splenic cDC2s and macrophages (Supplementary Fig. S7), but due to a low detection of SIINFEKL^+^ events in these two populations, along with a large variation in the data set, we could not draw any conclusions regarding the antigen-presentation. The data did however not seem to indicate an enhanced antigen presentation by cDC2s and macrophages as a result of MK062:TMX treatment. Additionally, we found that DCs in the tdLNs did not display increased antigen presentation after MK062:TMX treatment, indicating the spleen as the primary organ for antigen cross-presentation and T cell priming with this TAA-delivery platform (Supplementary Fig. S8).

Finally, we assessed the expression of the activation marker CD86 at 24 and 48 h after treatment and could confirm that liposomal TMX-201 activates splenic CD8α^+^ cDCs in vivo, evident by an upregulated expression of CD86 (Fig. 3E and supplementary figure S9). Together these results demonstrate that IV treatment with MK062:TMX liposomes induce significant activation and antigen presentation by APCs both in vitro and in vivo.

### The level of antigen presentation correlates with secretion of type I IFNs by pDCs

**Figure 4:**
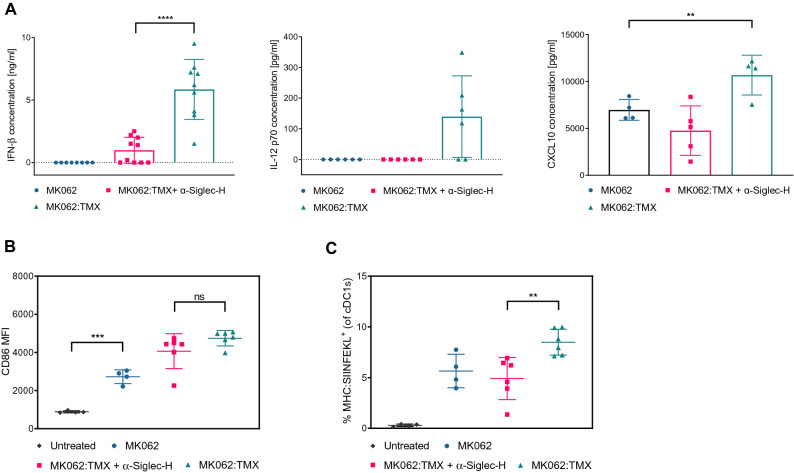
The level of antigen presentation by splenic CD8α^+^ cDCs after MK062:TMX delivery correlates with the secretion of IFN-β by pDCs. (**A**) Effect of pDC blocking on systemic levels of IFN-β, IL-12 p70 and IP-10. Serum levels of cytokines were measured by ELISA four hours after treatment. (**B**) Effect of pDC blocking on expression levels of activation marker CD86 expression by splenic CD8α^+^ cDCs. (**C**) Effect of pDC blocking on antigen presentation by splenic CD8α^+^ cDCs. Graphs depict data from one experiment that has been repeated with comparable results. Error bars in the graphs represent the mean ± SD. Differences were calculated with one-way ANOVA . *P ≤ 0.05, **P ≤ 0.01 ***P ≤ 0.001 ****P ≤ 0.0001.

IFN-α/β are produced in vast amounts by plasmacytoid DCs (pDCs) in response to TLR7 stimulation^47^. Based on the important role of type I interferons (IFN-α/β) for activation and antigen presenting capacities of DCs^48,49^, we evaluated the contribution of pDCs to the activating effect of TMX-201 by cell blocking experiments. To this end, one group of mice received an IV injection with MK062:TMX liposomes, while another group received MK062:TMX along with three intraperitoneal (IP) injections of α-Siglec-H antibody that functionally blocks pDCs. Blood samples were collected from the three groups of mice and serum was analyzed for the levels of IFN-β and the pro-inflammatory cytokines IP10 and IL-12 p70, that are also induced by innate immune stimulation. Our results demonstrate that MK062:TMX liposomes increase the systemic levels of both IFN-β, IP10 and IL-12 p70 and that functional pDC blocking reduced the systemic levels of all three cytokines (Fig. 4A). To evaluate if the secretion of pro-inflammatory cytokines was associated with enhanced activation and/or increased antigen-presentation by splenic CD8α^+^ cDC1s in response to MK062:TMX treatment, we did a flow cytometry analysis of spleens from the treated mice looking for activation and antigen presentation. The analysis did not show a difference in the activation of splenic CD8α^+^ cDCs when the pDCs were blocked (Fig. 4B) which indicates that CD8α^+^ cDC activation is not dependent on IFN-β stimulation. However, the frequency of splenic antigen presenting (MHC:SIINFEKL^+^) CD8α^+^ cDCs was significantly lower when the mice were treated with MK062:TMX and α-Siglec H (Fig. 4C). Notably, treatment with α-Siglec-H lowered the frequency of MHC:SIINFEKL^+^ CD8α^+^ cDCs to a level corresponding to treatment with MK062 liposomes without TLR7/8 agonist incorporated (Fig. 4C). This indicates that pDC blocking eliminates the enhancing effect that TMX-201 has on antigen presentation by cDC1s. Previous studies have demonstrated that type I IFNs promote intracellular antigen persistence and stimulates survival and activation of cross-presenting DCs resulting in enhanced cross-priming of CD8α^+^ T cells^50,51^, which made us speculate that there might be a link between the decreased amount of IFN-β and antigen presentation. However, we cannot conclude that the decreased antigen presentation observed in connection with pDC blocking is a direct result of the lower systemic level of IFN-β, since other cytokines could modulate the antigen presentation as well.

Despite previous reports suggesting that pDCs are capable of presenting antigen and priming T cells^52,53^, no SIINFEKL antigen presentation on splenic pDCs could be demonstrated in response to MK062:TMX treatment (supplementary Fig. S10). We did however observe an increase in the CD86 expression level, confirming the activation of pDCs upon treatment with MK062:TMX liposomes (supplementary Fig. S10).

### Liposomal antigen delivery efficiently expands adoptively transferred, minimally differentiated CD8 + T cells

**Figure 5:**
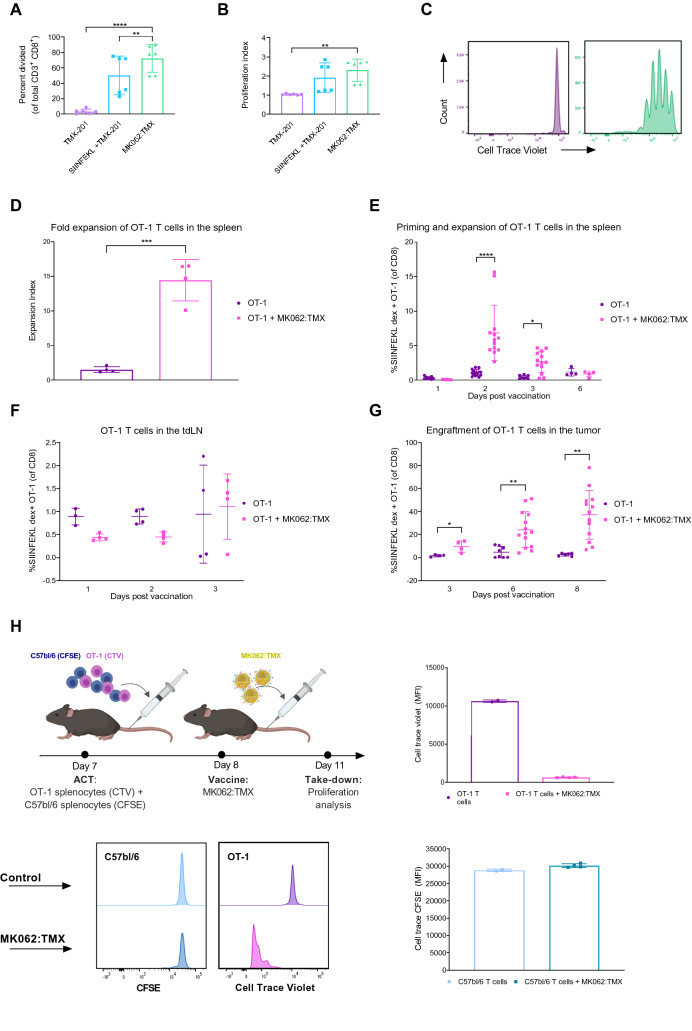
MK062:TMX liposomes induces proliferation of antigen-specific T cells in the spleen that subsequently home to the tumor. (**A**) Percentage of dividing antigen-specific CD8^+^ T cells addressed by Cell trace violet (CTV) dilution. (**B**) Proliferation index of OT-1 CD8^+^ T cells calculated in the software FlowJo V.10. (**C**) Representative CTV histograms from TMX-201-treated (left), or MK062:TMX-treated (right) samples. All conditions were tested in triplicates, in two independent experiments. **P ≤ 0.01, ****P ≤ 0.0001 (Tukey’s multiple comparison test). (**D**) Fold expansion calculated at day 2 following antigen-delivery. (**E**) Percentage of OT-1 T cells out of total CD8^+^ T cells in the spleen. (**F**) Percentage of OT-1 T cells of total CD8^+^ T cells in E.G7-OVA tumors at days 3, 6 and 8 following antigen-delivery. (**G**) Percentage of OT-1 T cells of total CD8^+^ T cells in the tdLN. Graphs represent 2–4 independent experiments (n = 4–6). *P ≤ 0.05 **P ≤ 0.01, ****P ≤ 0.0001 (Unpaired t test)). (**H**) Experimental set-up (upper left), representative histograms (lower left) and dilution of cell dye signals from OT-1 (upper right) or C57bl/6 CD8^+^ T cells (lower right).

Having established that MK062:TMX liposomes were capable of activating APCs and deliver antigen for presentation in vitro and in vivo, we evaluated the ability of the liposomal antigen delivery system to induce priming and expansion of CD8^+^ T cells. The activating properties of MK062:TMX-treated APCs were evaluated using an in vitro co-culture assay with MK062:TMX liposome-treated BMDCs and Cell Trace Violet (CTV) stained OT-1 T cells. Compared to liposomal adjuvant alone, treatment with MK062:TMX liposomes increased the proliferation and expansion index of OT-1 CD8^+^ T cells (Fig. 5A,B). Representative histograms showing the dilution of CTV signal used for calculation is shown for TMX-201 liposomes (Fig. 5C, left) and MK062:TMX liposomes (Fig. 5C, right).

Next, we wanted to evaluate the ability of MK062:TMX liposomes to induce priming and expansion of naïve OT-1 T cells in an in vivo tumor setting. For this purpose, female C57bl/6 mice were inoculated with E.G7-OVA and received IV infusions of naïve OT-1 T cells and MK062:TMX following the treatment schedule illustrated in Fig. 2A. Spleens, tdLNs and tumors were analyzed by flow cytometry on consecutive days following vaccination to assess OT-1 expansion and tumor engraftment. We found that MK062:TMX induced a significant proliferation of OT-1 T cells in the spleen, reaching 14.4-fold expansion 2 days after antigen delivery (Fig. 5D). This corresponded to an increased proportion of OT-1 cells in the spleen 2 and 3 days following antigen delivery (Fig. 5E). No notable proliferation of the OT-1 T cells could be observed in the tdLN (Fig. 5F), suggesting that priming was restricted to the spleen. In addition, subcutaneous delivery of antigen did not induce T cell engraftment in the spleen, even when administered at a 20-fold higher dose (Supplementary Fig. S11). At later time points, activated T cells entered the tumor reaching a level of up to 80% of the total CD8^+^ T cell population at day 8 following treatment (Fig. 5G). Notably, intratumoral engraftment of T cells coincided with the time point where the tumor volume is decreasing in response to therapy (Fig. 2B). It was also possible to expand two distinct T cell populations (OT-1 and pmel-1) simultaneously using a combination of MK062:TMX and MK098:TMX liposomes (Supplementary Fig. S12).

To address whether the proliferation observed for OT-1 CD8^+^ T cells was antigen specific we performed co-injection of CTV-stained OT-1 splenocytes and antigen-unspecific Cell Trace CFSE-stained splenocytes from C57bl/6 mice. Liposome antigen delivery was performed 24 h after ACT. Two days after liposome administration, the spleens were harvested to examine proliferation of the two stained splenocyte subsets. Encouragingly, the CTV signal was significantly reduced, indicating that OT-1 proliferation had been stimulated whereas the CFSE signal was equal to the controls (Fig. 5H). This demonstrate that MK062:TMX liposomes provide an antigen-specific T cell expansion.

### Tumor escape following ACT and antigen delivery cannot be prevented by additional T cell infusion, repeated peptide delivery or PD-1 checkpoint blockade

**Figure 6:**
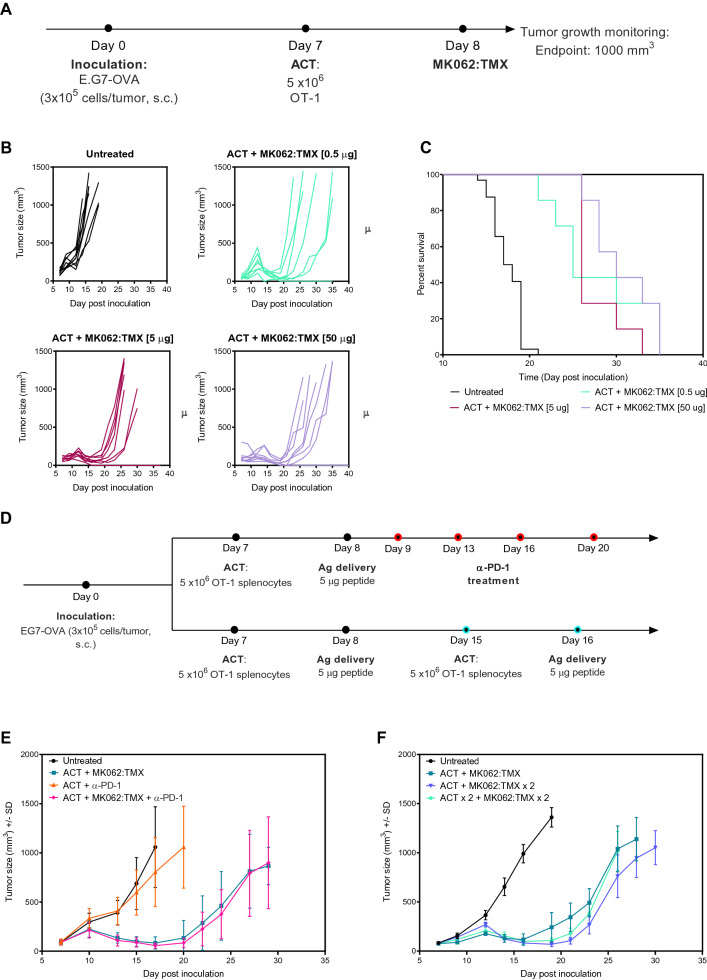
Tumors escape following treatment with ACT and antigen delivery. (**A**) Dosing schedule for efficacy studies. (**B**) Individual tumor growth curves. (**C**) Survival plots. (**D**) Dosing schedule for efficacy studies using either α-PD-1 treatment (top) or additional T cell infusion and/or antigen delivery (bottom) as interventions. (**E**) Tumor growth curves of mice treated with ACT + antigen delivery + α-PD-1. (**F**) Tumor growth curves of mice treated with two doses of ACT and/or antigen delivery.

To examine if the observed tumor escape could be prevented by treatment with a higher antigen dose, we performed a titration study with peptide doses ranging from 0.5 to 50 μg pr. dose (Fig. 6A). The doses were well tolerated but provided no additional therapeutic benefit (Fig. 6B,C).

It is well established that activated, tumor reactive T cells can become inactivated through checkpoint signaling, *e.g.* through interaction between PD-1 on T cells and PD-L1 expressed by cells in the TME^54^. Inactivation though engagement of PD-1 on transferred T cells could be a potential explanation for tumor regrowth after ACT. As anticipated, flow cytometric analysis of E.G7-OVA tumors demonstrated a high expression level of PD-1 on T cells following treatment (Supplementary Fig. S13). Addition of PD-1 checkpoint blockade to the treatment protocol did however not improve therapeutic efficacy in the E.G7-OVA tumors (Fig. 6D,E).

Considering that addition of α-PD-1 treatment provided no additional therapeutic benefit, we speculated if the relapse was associated with an absence of tumor reactive T cells at the time of tumor regrowth. To increase the number of tumor reactive T cells, an additional treatment with ACT infusion and/or antigen delivery was added to the original treatment schedule on day 15 and 16 after inoculation (Fig. 6D). However, the additional ACT infusion and/or antigen delivery did not affect the tumor growth or prevent tumor relapse after the initial response (Fig. 6F). These results indicate that the relapsed tumors had adapted PD-1-independent escape mechanisms to prevent the cytotoxic function of transferred T cells.

### Tumor relapse following ACT and TAA delivery is associated with the combined loss of antigen and downregulation of inflammatory pathways

**Figure 7:**
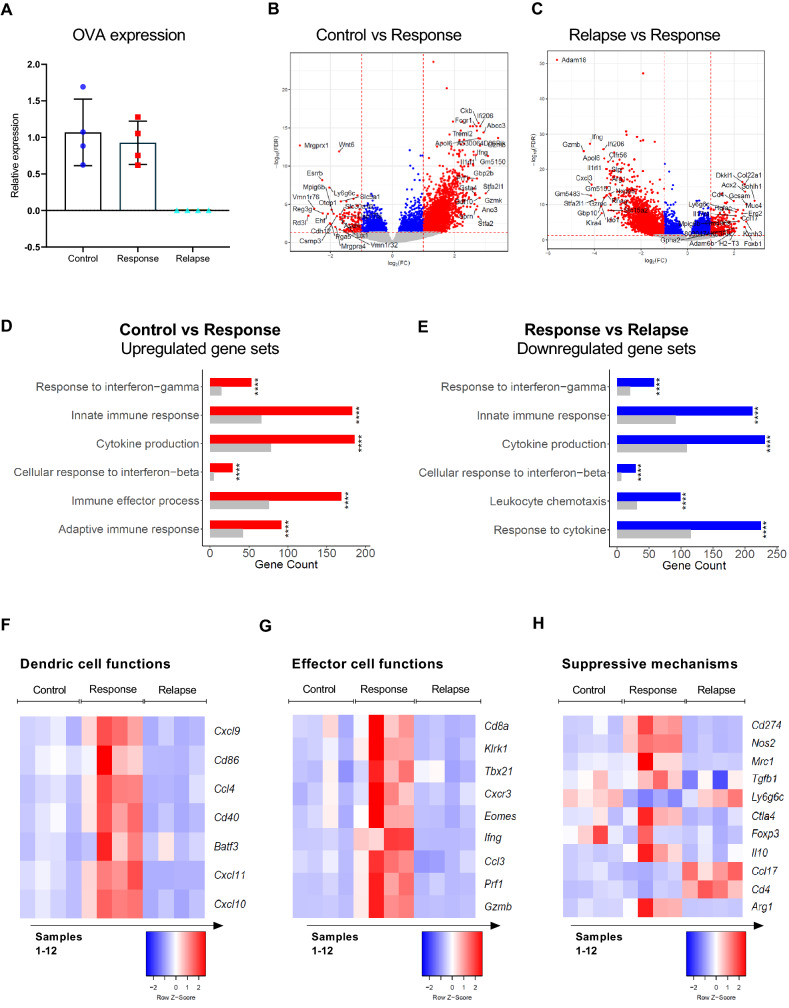
Responding tumors show expression of genes related to both inflammatory immune pathways, whereas relapsing tumors display a transcriptional profile associated with loss of target antigen and immune deprivation. (**A**) Comparative qPCR analysis of OVA expression in tumors. (**B-C**) Volcano plots illustrating differentially regulated genes (FDR < 0.05 and absolute log_2_FC > 1) between untreated control tumors and responsive tumors (**B**) and responsive tumors and relapsing tumors (**C**). Grey baseline: insignificant genes, blue dots: Significant downregulated genes above the log_2_FC threshold, red dots: Significant upregulated genes above the log_2_FC threshold, black dots: significant genes below the log_2_FC threshold. The 20 genes that show the most differentiated expression are annotated on each plot. (**D-E**) Gene ontology analysis illustrating upregulated gene sets (GO Terms) in (**D**) untreated tumors compared to treated, responding tumors and (**E**) downregulated genes in responsive tumors compared to relapsing tumors**.** Grey bars represent the number of up- or downregulated genes that one are expected to find by chance, whereas red and blue-colored bars show the number of genes that were found to be significantly up- or downregulated in respective samples. (**F–H**) Heatmaps depicting normalized (Z-score) RNAseq read counts of genes associated with dendritic cell functions (**F**)**,** effector cell functions (**G**) and immune suppressive mechanisms (**H**). Colors representing normalized gene expressions are row z-score normalized.

The progressive loss of anti-tumor activity of ACT cells could not be ameliorated by check-point inhibition or multiple dosing, which is consistent with previous observations made in preclinical studies^55,56^. Acquired resistance to ACT has been attributed to a progressive loss of antigen^36,57,58^ and downregulation of the target antigen OVA, which has been reported previously following OT-1 therapy^55^, could therefore provide a possible explanation behind the observed tumor escape. Expression of OVA in comparably sized tumors was therefore evaluated during the response and relapse growth phase (Supplementary Fig. S14**)**. Expression of OVA was found to be significantly downregulated in relapsing tumors compared to untreated control and responding tumors (Fig. 7A).

To further investigate the transcriptional changes associated with both the therapeutic response and the eventual relapse, RNA-seq was performed on E.G7-OVA tumors. Principle component analysis identified that control, responding and relapsing tumors separated to a reasonable extent (Supplementary Fig. S15). A clear difference between the gene expression profiles of responding and control tumors was evident by 2567 differently regulated genes (FDR < 0.05 and absolute log_2_FC > 1) (Fig. 7B). Between relapsing and responding tumors, a total of 3373 differently regulated genes were identified (FDR < 0.05 and absolute log_2_FC > 1) (Fig. 7C). When analyzing the top differentially expressed genes, we noted that immune-related genes were amongst the most prominent. Genes encoding Granzyme B and IFN-γ was markedly upregulated in responding tumors compared to both untreated and relapsing. This prompted us to perform a comparative gene ontology analysis (GO-TERM) to identify immune pathways in the different tumor samples. The GO-TERM analysis of responding tumors compared to untreated control tumors demonstrated that an inflammatory TME existed during the response phase. Specifically, genes associated with innate and adaptive immune responses, immune effector processes and cytokine production were upregulated (Fig. 7D). Consistent with this observation, the analysis showed that relapse was associated with downregulation of genes associated with e.g. leukocyte chemotaxis (CXCL9-11/CXCR3), innate immune response and IFN-β and IFN-γ production (Fig. 7E).

Further analysis of the transcriptional profiles revealed that tumors in the responding phase was associated with upregulated expression of genes related to DC activation, cytotoxic effector functions and immune suppressive cell subsets, whereas the same markers were largely absent from both untreated and relapsed tumors (Fig. 7F–H). Collectively these results indicated that tumor regression observed in response to ACT and liposomal antigen delivery was associated with infiltration of activated DCs, effector functions of cytotoxic CD8^+^ T cells and NK cells, but also expression of immune suppressive molecules from TAMs and upregulation of immune checkpoints, as has been previously observed^59^. This was an encouraging finding given that the absolute dose of TAA and TLR agonist reaching the tumor directly was very low (less than 1 ng for a ~ 100 μg tumor based on PET/CT data). Thus, the extensive repolarization of the TME indicated that the transferred T cells, after priming in the spleen, initiates a potent anti-tumor response that mobilizes multiple immune cell subsets even in the absence of prior lymphodepletion. During relapse from treatment, analysis of the target antigen and genes related to immune-infiltration predominately indicate that relapsing tumors becomes resistant to T cell based-therapy after the antigenic target is lost, and that this drives a reversal of the TME into an immune-suppressive state.

## Discussion

In order for immunological synapses to be established, tumor antigens and immune activating compounds must reach the same anatomical location as the DCs and T cells^60^. This realization has motivated therapeutic strategies that enhance delivery of TAA and adjuvant to the lymph nodes after subcutaneous administration. However, a large proportion of injected T cells reach the spleen via the same adhesion molecules that allow for trafficking to the LNs. In addition, delivery of antigen to the spleen has previously been demonstrated to facilitate T cell priming in response to vaccination both pre-clinically and clinically^29,30^. Given that a subcutaneous priming strategy do not reach the spleen to any significant extent, we decided to investigate systemic antigen delivery, specifically for combination with ACT.

Systemic delivery of tumor antigens and adjuvant using a liposome-based platform is a feasible strategy to prime previously non-activated, adoptively transferred T cells even at very low doses of TAA and adjuvant, and in the absence of lymphodepletion. The coordinated trafficking of T cells and liposomes to the spleen, as well as the brevity of liposome persistence, allow for a transient but highly effective priming of T cells. In our studies, we applied the TCR-transgenic T cell models pmel-1 and OT-1, using primarily non-activated T cells. While we believe that this experimental strategy showcase the potential of systemic T cell priming, it would be interesting to test the liposomal platform in combination with more therapeutically relevant T cell products such a T stem cell memory cells cultured with different cytokine cocktails or small molecule inhibitors. In our study, infusion of a pre-expanded and activated T cell population also showed therapeutic benefit in combination with the liposome delivery system. Thus, we envision that this technology is applicable for T cells which retain surface markers that allow them to enter lymphoid organs.

Antigen presentation was markedly improved when the tumor antigen liposomes were co-formulated with the TLR7/8 agonist TMX-201. Additionally, we identified a correlation between liposomal TMX-201 treatment and systemic levels of IFN-β. Functional blocking of pDCs, and a derived decrease in the systemic levels of IFN-β, correlated with a decrease in antigen presentation by splenic CD8α^+^ cDCs. Even though a direct causation between these events cannot be proved, the observations are supported by previous studies demonstrating that type I IFNs stimulate cross-presentation by DCs^30,48,49,51,61^. The provided TLR7/8 stimulation thereby ensures that pDCs are activated and supports CD8α^+^ cDC engagement for priming of tumor reactive CD8^+^ T cells.

The anti-tumor CD8^+^ T cell response in cancer patients is often curtailed by a suppressive tumor microenvironment, tolerance mechanisms and/or cytotoxic pretreatments^62^. Adoptive transfer of tumor-specific T cells can restore the T cell reactivity against tumors, but the treatment has also been reported to induce severe side effects in many patients. Toxicity caused by adoptively transferred T cells is often cytokine-mediated and can arise from unhinged expansion or on-target/off-tumor effects^63–65^. In addition, the routine use of high dose lymphodepleting chemotherapy prior to ACT limits patient eligibility and increases the risk of severe side-effects from treatment^66^.These risks pose a considerable barrier towards the broad application of ACT^3^. Here, we demonstrate that a single, very low systemic dose of tumor antigen and adjuvant using a novel delivery strategy can selectively expand and activate the transferred T-cells and induce regression of established tumors. The systemic antigen delivery platform is therefore promising for securing a specific and controlled expansion of tumor-reactive clones and thereby provides a refined immunotherapeutic approach.

## Materials and methods

### Study design

The study was designed to investigate if adoptively transferred naïve T cells can be primed in vivo by a liposomal antigen delivery consisting of the cognate antigen of the T cells co-formulated with a TLR7/8 agonist delivered systemically. Our hypothesis relied on the ability of the antigen delivery platform to prime, activate and expand transferred T cells by inducing antigen presentation and activation of APCs in the spleen. To explore this hypothesis, we conducted experiments using murine cell lines and primary cell cultures. We used mice as model organisms for our in vivo efficacy and mechanistic studies. The animal studies reported in this research article were approved by the Danish Animal Experiments Inspectorate and conducted in accordance with the ARRIVE guidelines. All methods were performed in accordance with the relevant guidelines and regulations as stated by The Danish Animal Experiments Inspectorate. For efficacy and mechanistic studies, mice were randomized according to tumor size and assigned to treatment groups by a blinded operator. Well-being of mice was monitored daily and they were weighed three times per week and were euthanized if they exhibited a weight loss exceeding 15%. None of the included mice displayed failure to thrive or excessive weight loss in the study. The tumor size was measured by a blinded operator using a digital caliper three times per week and mice were euthanized if the tumor size exceeded 1000 mm^3^, or if the tumor had ulcerated.

With respect to data inclusion/exclusion criteria or outliers, no data points were left out in any of the reported studies. All experiments have been performed with all relevant controls a minimum of two times. In some reported data sets, two or more experiments have been pooled. If a graph represents data points from pooled experiments, it is stated in the figure text. For efficacy studies, a minimum of 8 mice pr. treatment group was included. For in vitro and ex vivo studies, a range of 2–8 replicates were included for each individual study, depending on availability of sample material.

### Antigen synthesis and liposome formulation

All chemicals were purchased from Sigma-Aldrich, unless stated otherwise. Matrix-assisted laser desorption/ionization-time of flight (MALDI-TOF) mass spectrometry was performed on Bruker Autoflex TOF/TOF (Bruker Daltonics GmbH). Nuclear magnetic resonance (NMR) spectroscopy was carried out on a Bruker Ascend 400 (400 MHz for proton and 101 MHz for carbon) with resulting chemical shifts (δ) reported in parts per million (ppm) and coupling constants (J) in Hz. Semi-preparatory high-performance liquid chromatography (semi-prep HPLC) was performed on a Waters Semi-Preparative HPLC (Waters Corporation) equipped with a Waters Xterra^®^ C_8_ column (150 × 10 mm) using eluents (A) 0.1% TFA in water and (B) 0.1% TFA in acetonitrile. Analytical HPLC analyses were performed on a Shimadzu Nexera- × 2 UHPLC equipped with a Waters XBridge^®^ C_8_ column (5 μm, 4.6 × 150 mm) using the same eluent system at a flow rate of 1 mL/min. Liposome size, polydispersity, and zeta potential were analyzed by dynamic light scattering (DLS) using a Zetasizer Nano ZS (Malvern Panalytical Ltd). Liposome lipid concentrations were measured by quantifying ^31^P concentrations by inductively coupled plasma mass spectrometry (ICP-MS) on an iCAP Q ICP-MS system (Thermo Scientific). Samples were diluted 5000-fold in 2% HCl containing 10 ppb gallium as internal standard.

Antigens were synthesized according to the procedures in the supporting information (Supplementary Fig. S1).

Liposome formulation was carried out by lyophilizing tert-butanol:water (9:1 v/v) solutions of lipids (HSPC:Cholesterol:DSPE-mPEG_2000_ 57:38:5 mol%, Avanti Polar Lipids) with or without 2.5 mol% TLR7/8-agonist TMX-201 followed by rehydration at 70 °C with vortexing every 10 min during 1 h in HEPES buffer (25 mM HEPES, 150 mM NaCl, pH 7.4) to a lipid concentration of 50 mM. The multilamellar vesicles were downsized by extrusion through 2 × 100 nm polycarbonate filters at 70 °C with 10 repetitions on a pressure extruder. The liposomes were stored at 4 °C overnight before characterization. For antigen post-insertion, the liposomes were stirred at 45 °C while dropwise adding 10 mM solutions of cholesterol-linked peptide antigens in DMSO to an antigen concentration of 2.5 mol%. The solution was stirred for 15 h and then dialyzed against HEPES buffer for at least 12 h using Slide-A-Lyzer dialysis cassettes (10 k molecular weight cut-off, ThermoFischer Scientific). The antigen containing liposomal formulations were slowly filtered through a 0.45 µm nylon filter and characterized by size, polydispersity index and zeta potential by DLS, and determination of lipid, antigen, and adjuvant concentrations by ICP-MS and HPLC. Cryo-TEM was performed at the Center for Electron Nanoscopy at DTU.

### Cancer cell lines and tumor challenge in mice

The murine thymoma cell line E.G7-OVA was obtained from the American Type Culture Collection (ATCC, Manassas, VA CRL-2113) and the murine melanoma cell line B16-OVA was a kindly provided by Dr. Marianne Hokland, Aarhus University, Denmark. Both cell lines were maintained in RPMI 1640 medium supplemented with 10% FBS, 1% P/S and 0.4 mg/mL geneticin selective antibiotic (G418). Prior to tumor challenge, cells were harvested, washed and resuspended in complete RPMI 1640 medium.

### Biodistribution studies in mice

Mice were slowly injected via the tail vein with 100 μL radiolabeled liposomes containing ~ 25 MBq ^64^Cu. The radiolabeled liposomes were based on the same composition as the antigen-carrying liposomes but containing 1 mol% of DSPE-DOTA in order to chelate the Cu64. During the scan procedures mice were kept anaesthetized with 3% sevoflurane (Abbott) in 35%/65% O_2_/N_2_a. PET/CT imaging was performed and reconstructed as previously described^67^. PET/CT scans were performed 10 min, 2 h and 22 h after injection of radiolabeled liposomes. PET scans were performed with a 5 min acquisition time at the 10 min and 2 h scans and 15 min acquisition time at the 22 h scans. Regions of interest were manually drawn on PET/CT images using commercially available software (Inveon, Siemens Medical Systems, Malvern, PA, USA) and the activity of radiolabeled liposome reported as %ID/g for the indicated tissues.

After 48 h, animals were sacrificed by cervical dislocation and liver, spleen, tumor-draining lymph node, non-tumor-draining lymph node and tumor were harvest for quantification of radioactivity by gamma counting (Wizard Well Counter, Perkin Elmer, US). Weight corrected tissue activity was recorded using a decay and counting efficiency corrected protocol and reported as %ID/g.

### Efficacy studies in mice

Female C57BL/6 mice were obtained at 6 weeks of age from Janvier Labs (France) and housed at the Department of Experimental Medicine, University of Copenhagen. All procedure were approved by the National Animal Experiments Inspectorate. E.G7-OVA and B16-OVA tumors were established in C57BL/6 mice by *s.c.* injection of 3 × 10^5^ viable cells (day 0). Tumors were consequently allowed to establish for 7 days for E.G7-OVA and 10 days for B16-OVA before initiation of treatment. Before inclusion, mice were randomized according to tumor volume.

### Adoptive cell therapy using unstimulated cells and antigen delivery

Spleens were harvested from OT-1 and pmel-1 TCR transgenic mice after cervical dislocation, minced into small fragments and mechanically dispersed in 3–5 mL cold PBS. After filtering with a 70 μm cell strainer the cells were centrifuged and resuspended in lysis buffer to remove erythrocytes. Mice received a total dose of 5 × 10^6^ splenocytes, corresponding to ~ 0.5 × 10^6^ naïve CD8^+^ T cells. One day after adoptive cell transfer, mice were treated IV with peptide and/or liposomes.

### Adoptive cell therapy using in vitro expanded T cells

For splenic CD8^+^ T cell isolation, spleens were harvested from OT-1 TCR transgenic mice after cervical dislocation, minced into small fragments and mechanically dispersed in 3–5 mL cold PBS. After filtering with 70 μm cell strainer the cells were centrifuged and resuspended in lysis buffer to remove erythrocytes. Following wash in cold PBS, splenocytes were counted and resuspended in sterile phosphate buffer saline (PBS) containing 0.5% BSA and 0.1% NaN3 (FACS buffer). CD8^+^ T lymphocytes were purified using microbead isolation kits followed by magnetic-activated cell sorting (MACS) according to the manufacturer’s instructions (Miltenyi Biotec, Germany).

One day prior to culture, 6-well plates were coated with anti-CD3 (clone 2C11) and anti-CD28 (clone 37.51) antibodies (BioXcell) at a concentration of 5 µg/mL in sterile PBS at 4C. Isolated CD8^+^ T cells were plated in a-CD3/a-CD28 coated six-well-plates at a concentration of 1 × 10^6^ cells/mL in complete RPMI-1640 medium with 1% ITS solution. The following day (day 1), medium was supplemented with recombinant murine IL-2 [20 ng/mL] and IL-7 [5 ng/mL]. On day 2, cells were removed from the plate and washed in cold PBS, then resuspended in fresh medium containing IL-2 and IL-7. On day 3, cells were supplemented with fresh medium containing IL-21 [10 ng/mL]. On day 4, cells were counted, then washed and resuspended to a concentration of 50 × 10^6^ cells/mL. Recipient mice were injected IV in the tail vein with 100 µL cell suspension, corresponding to 5 × 10^6^ T cells.

### Statistics and data treatment

All statistics were done in in the Graph Pad Prism (8.1.1) software, except for the linear effects model, which was done using R. For survival analysis, log-rank test was performed. Difference in tumor sizes on individual days was analyzed using a linear mixed model, with a random effect for the mouse (subjectID), with fixed effect terms for the group, day and group × day interaction. Differences between groups were tested with one-way ANOVA followed by. Tukey’s post-test. For evaluation of expansion using Cell Trace Violet, the expansion index was calculated using the FlowJo V.10 software.

### In vitro antigen presentation assay

Bone marrow derived dendritic cells (BMDCs) were differentiated in vitro before antigen pulsing. Bone marrow cells were isolated from tibia and femur from C57bl/6 JrJ mice (Janvier). Bones were isolated and kept in tissue storage solution (MACS Miltenyi). After 2 min sterilization in 70% ethanol, bones were cut at each and flushed with medium. Following isolation, bone marrow cells were cultured in complete RPMI 1640 medium supplemented with 20 ng/mL mouse recombinant GM-CSF. On day 3, cells were supplemented with fresh medium containing GM-CSF. On day 6, immature BMDCs were harvested, re-plated and incubated with 1 µg/mL liposomal (MK062 or MK062:TMX) or free SIINFEKL peptide dissolved in PBS. BMDCs were harvested after 24–96 h in culture for flow cytometry analysis.

Two million cells/sample were washed with phosphate buffer saline (PBS) containing 0.5% BSA and 0.1% NaN3 (FACS buffer) and resuspended in Fc block (BD Bioscience). After blocking (5 min on ice), cells were stained with CD11c antibodies and assessed for antigen presentation by an antibody recognizing SIINFEKL presented on MHC I molecules (H-2 kb). Staining was done for 30 min at 4 °C. Subsequently, cells were washed, suspended in FACS buffer and subjected to flow cytometric analysis (BD LSR Fortessa X20).

### In vitro T cell proliferation assay

Bone marrow derived dendritic cells (BMDCs) were differentiated and treated with antigen as described above. For splenic CD8^+^ T cell isolation, spleens from OT-1 TCR transgenic mice were minced and mechanically dispersed in 3–5 mL cold PBS. After filtering with 70 μm cell strainer the cells were centrifuged and resuspended in lysis buffer to remove erythrocytes. Following wash in cold PBS, splenocytes were counted and resuspended in sterile PBS containing 0.5% BSA and 0.1% NaN3 (FACS buffer). CD8^+^ T lymphocytes were purified using microbead isolation kits followed by magnetic-activated cell sorting (MACS) according to the manufacturer’s guidelines (Miltenyi Biotec). Isolated CD8^+^ T cells were washed and stained with CellTrace™ Violet Cell Proliferation Kit (Thermo Fisher Scientific), by incubating cells at a density of 5 × 10^6^ cells/mL with 2.5 µM dye in warm PBS for 20 min at 37C following wash in 5× staining volume with complete medium.

For co-culture, 10^6^ BMDCs were plated with 3 × 10^6^ CD8^+^ T cells in 6 well flat bottom tissue culture plates in complete 1640 RMPI media with 1% ITS (Insulin-Transferrin-Selenium) solution. Following 4 day co-culture, the samples were harvested for flow cytometry analysis. Cells were washed with FACS buffer and resuspended in Fc block (BD Bioscience). After blocking for 5 min on ice, cells were stained with stained with fluorescent antibodies to visualize living, CD11c^-^, CD3^+^, CD8^+^ and Cell trace^+^ positive cells. Samples were analyzed on the BD Fortessa X-20 flow cytometer.

### Determination of systemic cytokine levels following liposome treatment

Blood was sampled from treated or untreated control female C57bl/6 mice from the sublingual vein, to determine systemic cytokine levels. Serum was prepared from the blood by centrifuging at 4 °C for 15 min at 2000*g* and cytokine levels were determined using mouse DuoSet ELISAs (R&D Systems). ELISAs were performed according to manufactures protocol.

### pDC blocking

pDC blocking was done with an IP injection of Siglec-H antibody (clone 440c, InVivoMab), 100 μg/dose for a total of 3 injections. The first two injections were given 24 h apart starting two days before antigen-delivery and the third injection was given immediately before liposomal antigen-delivery. Flow cytometry was used to confirm depletion of pDCs.

### Ex vivo analysis of antigen presentation

C57bl/6 female mice were treated with a single, IV injection of either soluble peptide [10 μg], liposomal adjuvant TMX-201, or the MK062 TMX formulation. 24 and 48 h after injection mice were sacrificed, and spleens were harvested for flow cytometry. Single cell suspensions were obtained by mechanical disruption and passing tissues through a 70 μM cell strainer. Ten million cells/sample were washed with PBS containing 0.5% BSA and 0.1% NaN3 (FACS buffer) and resuspended in Fc block. After blocking for 5 min on ice, cells were incubated with fluorochrome conjugated antibodies for 30 min at 4 °C. Subsequently, cells were washed, suspended in PBS and subjected to flow cytometric analysis (BD Fortessa). Splenic cDC1s were gated as CD45^+^, CD64^-^, CD26^+^, MHC II^hi^, CD11c^hi^, CD8a^+^, CD11b^-^, and antigen presentation was evaluated with an antibody recognizing SIINFEKL presented on MHC I (H-2 kb) molecules.

### Ex vivo analysis of T cell engraftment and proliferation

OT-1 TCR CD8^+^ T cells were harvested from TCR-trangenic ‘OT-1’ mice (C57BL/6-Tg(TcraTcrb)100Mjb/J) (Charles River) and stained with CellTrace™ Violet (CTV) Cell Proliferation Kit (Thermo Fisher Scientific) as described above.

E.G7-OVA bearing mice received 5 × 10^6^ splenocytes, corresponding to ~ 0.5 × 10^6^ naïve CD8^+^ T cells. One day after adoptive cell transfer, IV treatment with MK062:TMX liposomes was performed.

At multiple time points after liposome treatment, mice were sacrificed and organs were harvested for flow cytometry and evaluation of T cell proliferation and/or infiltration. Single cell suspensions were obtained from mouse organs by mechanical disruption (spleen) or enzymatic digestion (tumor). Ten million cells/sample were washed with PBS containing 0.5% BSA and 0.1% NaN3 (FACS buffer) and resuspended in Fc block. After blocking for 5 min on ice, cells were incubated with fluorochrome conjugated antibodies for 30 min at 4 °C. Subsequently, cells were washed, suspended in PBS and flow cytometry performed (BD Fortessa). The percentage and proliferation of antigen-specific, CD8 + T cells in the spleen and in the tumor was evaluated using a fluorescently labeled MHC multimer (ImmuDex) recognizing the SIINFEKL-specific T cells in combination with CTV.

### RNA isolation

Tumors were excised, snap frozen and stored at − 80 °C. Tumors were subsequently pulverized with the automated CryoPrep instrument (Covaris) and 10 mg was transferred to a CK mix bead beating tube (Bertin, France) with 350 µL RLT buffer (Qiagen) supplemented with 1% β-mercaptoethanol (Sigma). Tissue was homogenized in the Precellys 24 instrument (Bertin) 6500 rpm for 20 s. Samples were centrifuged for 3 min at 16,000*g* and the supernatant was collected. RNA was extracted using the RNeasy Mini kit (Qiagen) following the manufacturer’s guidelines. RNA concentration was measured with the NanoDrop One (Thermo Scientific) and RNA integrity confirmed with the 2100 Bioanalyzer RNA Nano Chip (Agilent).

### qPCR analysis of OVA expression

cDNA was prepared from 300 ng RNA with the Affinity script cDNA synthesis kit (Agilent) according to the manufacturer’s instructions with 0.245 µg oligo(dT) primers and 0.55 µg random primers. Comparative quantitation qPCR of chicken ovalbumin was performed with TaqMan primers Gg03366807_m1 (Thermo Fischer Scientific) and the TaqMan fast advanced master mix (Thermo Fischer Scientific) according to the manufacturer’s guidelines. Ct values were normalized to the housekeeping genes ppih (Mm01188566_g1, Thermo Fischer Scientific) and sdha (Mm01352366_m1, Thermo Fischer Scientific) in the qBase + (Biogazelle) software and expression levels for the response and relapse groups were normalized to mean expression levels of control.

### RNA-seq

For RNA library preparation, the RNA of tumors was diluted to the identical concentration with RNase-free water. Four biological replicates were generated for each group. Library construction was conducted following the standardized procedure at Beijing Genome Institute (BGI, Copenhagen, Denmark). Pair-end reads of 100 bp read-length were sequenced on the BGISEQ-500RS sequencer. Qualified raw data were provided by the Beijing Genomics Institute in FASTQ format.

### Preprocessing of raw data Fastq to count data

RNA-seq quantification was performed using the bcbio-nextgen pipeline including state-of-the-art bioinformatics tools. This included (1) quality assessment of the fastq files by using fastq, (2) alignment to the mm10 mouse genome reference by using hisat2, and (3) counting of reads by using featureCounts.

### DEA

Differential expression analysis (DEA) of coding genes was performed by using the DESeq2 R package. Non-coding genes in the quantified RNA-seq data was filtered out using the biomaRt R package followed by library size normalization and DEA. In total, two sets of analyses were made: treatment against control and relapse against treatment. Post-hoc filtering was applied to the DEA results (absolute log2FC > 1 and FDR < 0.05).

### GO enrichment

GO enrichment was performed by using the topGO R package. Significant genes were separated based on the log2FC direction. All genes with accessible P values from the DEA (14,178 genes) were used as genetic background for the GO enrichment test. Subsequent FDR correction was performed on the GO enrichment results.

### Heatmaps

Quantified gene expression data was normalized for library size using the estimateSizeFactorsForMatrix function in the DESeq2 R package. Genes of interest that were shown to be significantly different among the groups (abs(log2FC) > 1 and FDR < 0.05) were visualized by using the heatmap.2 function from the gplots R package. Row z-score normalization was performed to standardize colors between genes for comparison of up- and down-regulated genes.

## Supplementary Information

Supplementary Information.

## Data Availability

All data needed to evaluate the conclusions in the paper are present in the paper or the Supplementary Materials.

## References

[1] Yang Y (2015). Cancer immunotherapy: Harnessing the immune system to battle cancer. J. Clin. Invest..

[2] Scheper W, Kelderman S, Fanchi LF, Linnemann C, Bendle G, de Rooij MAJ, Hirt C, Mezzadra R, Slagter M, Dijkstra K, Kluin RJC, Snaebjornsson P, Milne K, Nelson BH, Zijlmans H, Kenter G, Voest EE, Haanen JBAG, Schumacher TN (2019). Low and variable tumor reactivity of the intratumoral TCR repertoire in human cancers. Nat. Med..

[3] Johnson LA, June CH (2017). Driving gene-engineered T cell immunotherapy of cancer. Cell Res..

[4] Sharma P, Hu-Lieskovan S, Wargo JA, Ribas A (2017). Primary, adaptive, and acquired resistance to cancer immunotherapy. Cell.

[5] Ghoneim HE, Zamora AE, Thomas PG, Youngblood BA (2016). Cell-intrinsic barriers of T cell-based immunotherapy. Trends Mol. Med..

[6] Shah NN, Fry TJ (2019). Mechanisms of resistance to CAR T cell therapy. Nat. Rev. Clin. Oncol..

[7] Gattinoni L, Klebanoff CA, Palmer DC, Wrzesinski C, Kerstann K, Yu Z, Finkelstein SE, Theoret MR, Rosenberg SA, Restifo NP (2005). Acquisition of full effector function in vitro paradoxically impairs the in vivo antitumor efficacy of adoptively transferred CD8+ T cells. J. Clin. Invest..

[8] Klebanoff CA, Gattinoni L, Torabi-Parizi P, Kerstann K, Cardones AR, Finkelstein SE, Palmer DC, Antony PA, Hwang ST, Rosenberg SA, Waldmann TA, Restifo NP (2005). Central memory self/tumor-reactive CD8+ T cells confer superior antitumor immunity compared with effector memory T cells. Proc. Natl. Acad. Sci..

[9] Pilipow K, Scamardella E, Puccio S, Gautam S, De Paoli F, Mazza EM, De Simone G, Polletti S, Buccilli M, Zanon V, Di Lucia P, Iannacone M, Gattinoni L, Lugli E (2018). Antioxidant metabolism regulates CD8+ T memory stem cell formation and antitumor immunity. JCI Insight.

[10] Kondo T, Morita R, Okuzono Y, Nakatsukasa H, Sekiya T, Chikuma S, Shichita T, Kanamori M, Kubo M, Koga K, Miyazaki T, Kassai Y, Yoshimura A (2017). Notch-mediated conversion of activated T cells into stem cell memory-like T cells for adoptive immunotherapy. Nat. Commun..

[11] Minagawa A, Yoshikawa T, Yasukawa M, Hotta A, Kunitomo M, Iriguchi S, Takiguchi M, Kassai Y, Imai E, Yasui Y, Kawai Y, Zhang R, Uemura Y, Miyoshi H, Nakanishi M, Watanabe A, Hayashi A, Kawana K, Fujii T, Nakatsura T, Kaneko S (2018). Enhancing T cell receptor stability in rejuvenated iPSC-derived T cells improves their use in cancer immunotherapy. Cell Stem Cell.

[12] Vizcardo R, Masuda K, Yamada D, Ikawa T, Shimizu K, Fujii SI, Koseki H, Kawamoto H (2013). Regeneration of human tumor antigen-specific T cells from iPSCs derived from mature CD8+ T cells. Cell Stem Cell.

[13] Zhou J, Shen X, Huang J, Hodes RJ, Rosenberg SA, Robbins PF (2005). Telomere length of transferred lymphocytes correlates with in vivo persistence and tumor regression in melanoma patients receiving cell transfer therapy. J. Immunol..

[14] Franssen LE, Roeven MWH, Hobo W, Doorn R, Oostvogels R, Falkenburg JHF, Van De Donk NW, Kester MGD, Fredrix H, Westinga K, Slaper-Cortenbach I, Spierings E, Kersten MJ, Dolstra H, Mutis T, Schaap N, Lokhorst HM (2017). A phase I/II minor histocompatibility antigen-loaded dendritic cell vaccination trial to safely improve the efficacy of donor lymphocyte infusions in myeloma. Bone Marrow Transplant..

[15] Rapoport AP, Aqui NA, Stadtmauer EA, Vogl DT, Fang H-B, Cai L, Janofsky S, Chew A, Storek J, Akpek G, Badros A, Yanovich S, Tan MT, Veloso E, Pasetti MF, Cross A, Philip S, Murphy H, Bhagat R, Zheng Z, Milliron T, Cotte J, Cannon A, Levine BL, Vonderheide RH, June CH (2011). Combination immunotherapy using adoptive T-cell transfer and tumor antigen vaccination on the basis of hTERT and survivin after ASCT for myeloma. Blood.

[16] Rapoport AP, Aqui NA, Stadtmauer EA, Vogl DT, Xu YY, Kalos M, Cai L, Bin Fang H, Weiss BM, Badros A, Yanovich S, Akpek G, Tsao P, Cross A, Mann D, Philip S, Kerr N, Brennan A, Zheng Z, Ruehle K, Milliron T, Strome SE, Salazar AM, Levine BL, June CH (2014). Combination immunotherapy after asct for multiple myeloma using MAGE-A3/Poly-ICLC immunizations followed by adoptive transfer of vaccine-primed and costimulated autologous T cells. Clin. Cancer Res..

[17] Wang X, Wong CW, Urak R, Mardiros A, Budde LE, Chang WC, Thomas SH, Brown CE, La Rosa C, Diamond DJ, Jensen MC, Nakamura R, Zaia JA, Forman SJ (2015). CMVpp65 vaccine enhances the antitumor efficacy of adoptively transferred CD19-redirected CMV-specific T cells. Clin. Cancer Res..

[18] Gnjatic S, Bhardwaj N (2013). Antigen depots: T cell traps?. Nat. Med..

[19] Hailemichael Y, Dai Z, Jaffarzad N, Ye Y, Medina MA, Huang X-F, Dorta-Estremera SM, Greeley NR, Nitti G, Peng W, Liu C, Lou Y, Wang Z, Ma W, Rabinovich B, Schluns KS, Davis RE, Hwu P, Overwijk WW (2013). Persistent antigen at vaccination sites induces tumor-specific CD8+ T cell sequestration, dysfunction and deletion. Nat. Med..

[20] Segal G, Prato S, Zehn D, Mintern JD, Villadangos JA (2016). Target density, not affinity or avidity of antigen recognition, determines adoptive T cell therapy outcomes in a mouse lymphoma model. J. Immunol..

[21] Zahm CD, Colluru VT, McNeel DG (2017). Vaccination with high-affinity epitopes impairs antitumor efficacy by increasing PD-1 expression on CD8 + T cells. Cancer Immunol. Res..

[22] Liu H, Moynihan KD, Zheng Y, Szeto GL, Li AV, Huang B, Van Egeren DS, Park C, Irvine DJ (2014). Structure-based programming of lymph-node targeting in molecular vaccines. Nature.

[23] Kuai R, Ochyl LJ, Bahjat KS, Schwendeman A, Moon JJ (2017). Designer vaccine nanodiscs for personalized cancer immunotherapy. Nat. Mater..

[24] Muraoka D, Harada N, Hayashi T, Tahara Y, Momose F, Sawada SI, Mukai SA, Akiyoshi K, Shiku H (2014). Nanogel-based immunologically stealth vaccine targets macrophages in the medulla of lymph node and induces potent antitumor immunity. ACS Nano.

[25] Luo M, Wang H, Wang Z, Cai H, Lu Z, Li Y, Du M, Huang G, Wang C, Chen X, Porembka MR, Lea J, Frankel AE, Fu YX, Chen ZJ, Gao J (2017). A STING-activating nanovaccine for cancer immunotherapy. Nat. Nanotechnol..

[26] Ma L, Dichwalkar T, Chang JYH, Cossette B, Garafola D, Zhang AQ, Fichter M, Wang C, Liang S, Silva M, Kumari S, Mehta NK, Abraham W, Thai N, Li N, Wittrup KD, Irvine DJ (2019). Enhanced CAR-T cell activity against solid tumors by vaccine boosting through the chimeric receptor. Science.

[27] Munn DH, Mellor AL (2006). The tumor-draining lymph node as an immune-privileged site. Immunol. Rev..

[28] Parisi G, Saco JD, Salazar FB, Tsoi J, Krystofinski P, Puig-Saus C, Zhang R, Zhou J, Cheung-Lau GC, Garcia AJ, Grasso CS, Tavaré R, Hu-Lieskovan S, Mackay S, Zalevsky J, Bernatchez C, Diab A, Wu AM, Comin-Anduix B, Charych D, Ribas A (2020). Persistence of adoptively transferred T cells with a kinetically engineered IL-2 receptor agonist. Nat. Commun..

[29] Sahin U, Derhovanessian E, Miller M, Kloke B-P, Simon P, Löwer M, Bukur V, Tadmor AD, Luxemburger U, Schrörs B, Omokoko T, Vormehr M, Albrecht C, Paruzynski A, Kuhn AN, Buck J, Heesch S, Schreeb KH, Müller F, Ortseifer I, Vogler I, Godehardt E, Attig S, Rae R, Breitkreuz A, Tolliver C, Suchan M, Martic G, Hohberger A, Sorn P, Diekmann J, Ciesla J, Waksmann O, Brück A-K, Witt M, Zillgen M, Rothermel A, Kasemann B, Langer D, Bolte S, Diken M, Kreiter S, Nemecek R, Gebhardt C, Grabbe S, Höller C, Utikal J, Huber C, Loquai C, Türeci Ö (2017). Personalized RNA mutanome vaccines mobilize poly-specific therapeutic immunity against cancer. Nature.

[30] Kranz LM, Diken M, Haas H, Kreiter S, Loquai C, Reuter KC, Meng M, Fritz D, Vascotto F, Hefesha H, Grunwitz C, Vormehr M, Hüsemann Y, Selmi A, Kuhn AN, Buck J, Derhovanessian E, Rae R, Attig S, Diekmann J, Jabulowsky RA, Heesch S, Hassel J, Langguth P, Grabbe S, Huber C, Türeci Ö, Sahin U (2016). Systemic RNA delivery to dendritic cells exploits antiviral defence for cancer immunotherapy. Nature.

[31] Shimizu T, Abu Lila AS, Kawaguchi Y, Shimazaki Y, Watanabe Y, Mima Y, Hashimoto Y, Okuhira K, Storm G, Ishima Y, Ishida T (2018). A novel platform for cancer vaccines: Antigen-selective delivery to splenic marginal zone B cells via repeated injections of PEGylated liposomes. J. Immunol..

[32] Weninger W, Crowley MA, Manjunath N, von Andrian UH (2001). Migratory properties of naive, effector, and memory CD8+ T cells. J. Exp. Med..

[33] Børresen B, Henriksen JR, Clergeaud G, Jørgensen JS, Melander F, Elema DR, Szebeni J, Engelholm SA, Kristensen AT, Kjær A, Andresen TL, Hansen AE (2018). Theranostic imaging may vaccinate against the therapeutic benefit of long circulating PEGylated liposomes and change cargo pharmacokinetics. ACS Nano.

[34] Baruch EN, Berg AL, Besser MJ, Schachter J, Markel G (2017). Adoptive T cell therapy: An overview of obstacles and opportunities. Cancer.

[35] Hirosue S, Kourtis IC, van der Vlies AJ, Hubbell JA, Swartz MA (2010). Antigen delivery to dendritic cells by poly(propylene sulfide) nanoparticles with disulfide conjugated peptides: Cross-presentation and T cell activation. Vaccine.

[36] Majzner RG, Mackall CL (2018). Tumor antigen escape from car t-cell therapy. Cancer Discov..

[37] Maeurer MJ, Gollin SM, Martin D, Swaney W, Bryant J, Castelli C, Robbins P, Parmiani G, Storkus WJ, Lotze MT (1996). Tumor escape from immune recognition: Lethal recurrent melanoma in a patient associated with downregulation of the peptide transporter protein TAP-1 and loss of expression of the immunodominant MART-1/Melan-A antigen. J. Clin. Invest..

[38] Duffner U, Abdel-Mageed A, Younge J, Tornga C, Scott K, Staddon J, Elliott K, Stumph J, Kidd P (2016). The possible perils of targeted therapy. Leukemia.

[39] Moynihan KD, Opel CF, Szeto GL, Tzeng A, Zhu EF, Engreitz JM, Williams RT, Rakhra K, Zhang MH, Rothschilds AM, Kumari S, Kelly RL, Kwan BH, Abraham W, Hu K, Mehta NK, Kauke MJ, Suh H, Cochran JR, Lauffenburger DA, Wittrup KD, Irvine DJ (2016). Eradication of large established tumors in mice by combination immunotherapy that engages innate and adaptive immune responses. Nat. Med..

[40] Kaluza KM, Kottke T, Diaz RM, Rommelfanger D, Thompson J, Vile R (2012). Adoptive transfer of cytotoxic T lymphocytes targeting two different antigens limits antigen loss and tumor escape. Hum. Gene Ther..

[41] Khong H, Volmari A, Sharma M, Dai Z, Imo CS, Hailemichael Y, Singh M, Moore DT, Xiao Z, Huang X, Horvath TD, Hawke DH, Overwijk WW (2018). Peptide vaccine formulation controls the duration of antigen presentation and magnitude of tumor-specific CD8 ^+^ T cell response. J. Immunol..

[42] Blair DA, Turner DL, Bose TO, Pham Q-M, Bouchard KR, Williams KJ, McAleer JP, Cauley LS, Vella AT, Lefrancois L (2011). Duration of antigen availability influences the expansion and memory differentiation of T cells. J. Immunol..

[43] Obst R, van Santen H-M, Malemed R, Kamphorst AO, Benoist C, Mathis D (2007). Sustained antigen presentation can promote an immunogenic T cell response, like dendritic cell activation. PNAS.

[44] Hildner K, Edelson BT, Purtha WE, Diamond M, Matsushita H, Kohyama M, Calderon B, Schraml BU, Unanue ER, Diamond MS, Schreiber RD, Murphy TL, Murphy KM (2008). Batf3 deficiency reveals a critical role for CD8α+ dendritic cells in cytotoxic T cell immunity. Science (8–).

[45] Spranger S, Dai D, Horton B, Gajewski TF (2017). Tumor-residing Batf3 dendritic cells are required for effector T cell trafficking and adoptive T cell therapy. Cancer Cell.

[46] Broz ML, Binnewies M, Boldajipour B, Nelson AE, Pollack JL, Erle DJ, Barczak A, Rosenblum MD, Daud A, Barber DL, Amigorena S, van’T Veer LJ, Sperling AI, Wolf DM, Krummel MF (2014). Dissecting the tumor myeloid compartment reveals rare activating antigen-presenting cells critical for T cell immunity. Cancer Cell.

[47] Gibson SJ, Lindh JM, Riter TR, Gleason RM, Rogers LM, Fuller AE, Oesterich JAL, Gorden KB, Qiu X, McKane SW, Noelle RJ, Miller RL, Kedl RM, Fitzgerald-Bocarsly P, Tomai MA, Vasilakos JP (2002). Plasmacytoid dendritic cells produce cytokines and mature in response to the TLR7 agonists, imiquimod and resiquimod. Cell. Immunol..

[48] Diamond MS, Kinder M, Matsushita H, Mashayekhi M, Dunn GP, Archambault JM, Lee H, Arthur CD, White JM, Kalinke U, Murphy KM, Schreiber RD (2011). Type I interferon is selectively required by dendritic cells for immune rejection of tumors. J. Exp. Med..

[49] Oh JZ, Kurche JS, Burchill MA, Kedl RM (2011). TLR7 enables cross-presentation by multiple dendritic cell subsets through a type I IFN-dependent pathway. Blood.

[50] Le Bon A, Etchart N, Rossmann C, Ashton M, Hou S, Gewert D, Borrow P, Tough DF (2003). Cross-priming of CD8+ T cells stimulated by virus-induced type I interferon. Nat. Immunol..

[51] Lorenzi S, Mattei F, Sistigu A, Bracci L, Spadaro F, Sanchez M, Spada M, Belardelli F, Gabriele L, Schiavoni G (2011). Type I IFNs control antigen retention and survival of CD8 + dendritic cells after uptake of tumor apoptotic cells leading to cross-priming. J. Immunol..

[52] Mouriès J, Moron G, Schlecht G, Escriou N, Dadaglio G, Lederc C (2008). Plasmacytoid dendritic cells efficiently cross-prime naive T cells in vivo after TLR activation. Blood.

[53] Tel J, Schreibelt G, Sittig SP, Mathan TSM, Buschow SI, Cruz LJ, Lambeck AJA, Figdor CG, De Vries IJM (2013). Human plasmacytoid dendritic cells efficiently cross-present exogenous Ags to CD8+ T cells despite lowerAg uptake than myeloid dendritic cell subsets. Blood.

[54] Ahmadzadeh M, Johnson LA, Heemskerk B, Wunderlich JR, Dudley ME, White DE, Rosenberg SA (2008). Tumor antigen-specific CD8 T cells infiltrating the tumor express high levels of PD-1 and are functionally impaired. Blood.

[55] Knutson DL, Vile RG (2014). Adoptive T cell therapy promotes the emergence of genomically altered tumor escape variants. Int. J. Cancer.

[56] Wylie B, Chee J, Forbes CA, Booth M, Stone SR, Buzzai A, Abad A, Foley B, Cruickshank MN, Waithman J (2019). Acquired resistance during adoptive cell therapy by transcriptional silencing of immunogenic antigens. Oncoimmunology.

[57] O’Rourke DM, Nasrallah MP, Desai A, Melenhorst JJ, Mansfield K, Morrissette JJD, Martinez-Lage M, Brem S, Maloney E, Shen A, Isaacs R, Mohan S, Plesa G, Lacey SF, Navenot JM, Zheng Z, Levine BL, Okada H, June CH, Brogdon JL, Maus MV (2017). A single dose of peripherally infused EGFRvIII-directed CAR T cells mediates antigen loss and induces adaptive resistance in patients with recurrent glioblastoma. Sci. Transl. Med..

[58] Garrido F, Aptsiauri N, Doorduijn EM, GarciaLora AM, van Hall T (2016). The urgent need to recover MHC class I in cancers for effective immunotherapy. Curr. Opin. Immunol..

[59] Spranger S, Spaapen RM, Zha Y, Williams J, Meng Y, Ha TT, Gajewski TF (2013). Up-regulation of PD-L1, IDO, and Tregs in the melanoma tumor microenvironment is driven by CD8+ T cells. Sci. Transl. Med..

[60] Schmidt ST, Khadke S, Korsholm KS, Perrie Y, Rades T, Andersen P, Foged C, Christensen D (2016). The administration route is decisive for the ability of the vaccine adjuvant CAF09 to induce antigen-specific CD8+T-cell responses: The immunological consequences of the biodistribution profile. J. Control Release.

[61] G. Schiavoni, G., Mattei, F. & Gabriele, L. Type I interferons as stimulators of DC-mediated cross-priming: Impact on anti-tumor response. *Front. Immunol.* **4** (2013) (available at www.frontiersin.org). 10.3389/fimmu.2013.00483 PMC3872318 24400008

[62] Das RK, Vernau L, Grupp SA, Barrett DM (2019). Naïve T-cell deficits at diagnosis and after chemotherapy impair cell therapy potential in pediatric cancers. Cancer Discov..

[63] Sultan H, Trillo-Tinoco J, Rodriguez P, Celis E (2017). Effective antitumor peptide vaccines can induce severe autoimmune pathology. Oncotarget.

[64] Jin Z, Xiang R, Qing K, Li X, Zhang Y, Wang L, Zhu H, Mao Y, Xu Z, Li J (2018). The severe cytokine release syndrome in phase I trials of CD19-CAR-T cell therapy: A systematic review. Ann. Hematol..

[65] Norelli M, Camisa B, Barbiera G, Falcone L, Purevdorj A, Genua M, Sanvito F, Ponzoni M, Doglioni C, Cristofori P, Traversari C, Bordignon C, Ciceri F, Ostuni R, Bonini C, Casucci M, Bondanza A (2018). Monocyte-derived IL-1 and IL-6 are differentially required for cytokine-release syndrome and neurotoxicity due to CAR T cells. Nat. Med..

[66] Lowe KL, MacKall CL, Norry E, Amado R, Jakobsen BK, Binder G (2018). Fludarabine and neurotoxicity 
in engineered T-cell therapy. Gene Ther..

[67] Jensen AI, Severin GW, Hansen AE, Fliedner FP, Eliasen R, Parhamifar L, Kjær A, Andresen TL, Henriksen JR (2018). Remote-loading of liposomes with manganese-52 and in vivo evaluation of the stabilities of 52Mn-DOTA and 64Cu-DOTA using radiolabelled liposomes and PET imaging. J. Control Release.

