# Supplementary material for: Enhancing adoptive CD8 T cell therapy by systemic delivery of tumor associated antigens
Source: Sci Rep. 2021 Oct 5;11:19794. doi: 10.1038/s41598-021-99347-0 (PMC8492729; doi:10.1038/s41598-021-99347-0)
Supplement: Supplementary file 1 — Supplementary Information. [file 41598_2021_99347_MOESM1_ESM.pdf]

Supplementary Information

Supplementary Materials (S1)

Procedures for antigen synthesis

Peptides synthesis was carried out on a Biotage Initiator+ Alstra automated microwave peptide synthesizer using standard Fmoc chemistry and reagents purchased from Iris Biotech Gmbh as follows: Peptides were synthesized on preloaded Fmoc-protected wang resins with standard Fmoc-protected amino acids, HATU, and 2,4,6-trimethylpyridine in N,N-dimethylformamide (DMF) at room temperature for 45 min or at 75°C for 5 min per coupling. Deprotections were carried out in 20% Piperidine in DMF (v/v). Cleavage from the resin was carried out in trifluoroacetic acid/triisopropylsilane/1,2-ethanedithiol/water 94:2:2:2 (v/v), followed by precipitation in diethyl ether and purification by semi-preparatory high-performance liquid chromatography.

(a) *Cholesterol-SH*: Cysteamine (1000 mg, 12.96 mmol) was dissolved in tetrahydrofuran (40 mL), then cooled to 4°C. Cholesteryl chloroformate (3894 mg, 8.67 mmol) was dissolved in tetrahydrofuran (15 mL) and added 2,4,6-trimethylpyridine (1150 µL, 8.70 mmol). This mixture was then added to the solution of cysteamine to form a white mixture that was refluxed for 30 min. The mixture was then filtered, evaporated onto Celite, and purified by dry column vacuum chromatography (hexane/chloroform/ethyl acetate 80:0:0 → 30:50:5). Yielded 3635 mg (86%) as a white solid. R_f = 0.34 (hexane/chloroform/ethyl acetate 20:20:1). MALDI-TOF MS: Calculated mass of $C_{30}H_{51}NO_2S$ = 489.36. Observed $[M+Na]^+$ as m/z = 512.19. ^{1}H NMR (400 MHz, Chloroform-d) δ 5.37 (dt, *J* = 5.4, 2.0 Hz, 1H), 5.00 (s, 1H), 4.50 (dt, *J* = 11.4, 6.3 Hz, 1H), 3.35 (q, *J* = 6.3 Hz, 2H), 2.66 (dt, *J* = 8.5, 6.5 Hz, 2H), 2.41 - 2.18 (m, 2H), 2.04 - 1.79 (m, 5H), 1.64 - 0.79 (m, 34H), 0.67 (s, 3H). ^{13}C NMR (101 MHz, $CDCl_3$) δ 156.1, 139.9, 122.7, 74.7, 56.8, 56.3, 50.1, 44.0, 42.5, 39.9, 39.7, 38.7, 37.1, 36.7, 36.3, 35.9, 32.1, 32.0, 28.4, 28.3, 28.2, 25.2, 24.4, 24.0, 23.0, 22.7, 21.2, 19.5, 18.9, 12.0.

(b) *MK062 and MK098*: Synthesized peptides (CSIINFEKL for *MK062*; CKVPRQDWL for *MK098*) were dissolved in NMP to approximately 0.01M, then cooled to 4°C before a 0.15M solution of 4,4′-dipyridyl disulfide (1.25 eq) in NMP was added to the peptide solution. This mixture was stirred at 4°C under nitrogen for 15 minutes before a 0.15M solution of *Cholesterol-SH* (1.25 eq) in NMP was added. The crude product was precipitated from NMP in diethyl ether and the resulting white solids purified by semi-prep HPLC. *MK062:* Yielded 78 mg (37%). HPLC (>95%). MALDI-TOF MS: Calculated mass of $C_{78}H_{128}N_{12}O_{16}S_2$ = 1552.90. Observed $[M+Na]^+$ as m/z = 1575.89; and $[M-H+2Na]^+$ as m/z = 1597.89. *MK098:* Yielded 56.9 mg (35%). HPLC (>95%). MALDI-TOF MS: Calculated mass of $C_{85}H_{136}N_{18}O_{17}S_2$ = 1744.98. Observed $[M+H]^+$ as m/z = 1746.15; and $[M+Na]^+$ as m/z = 1768.21.

(c) *Cholesterol-VS*: Divinyl sulfone (265 µL, 2.64 mmol) was dissolved in dichloromethane (10 mL) and added triethylamine (300 µL, 2.15 mmol) before a solution of *Cholesterol-SH* (1078 mg, 2.20 mmol) in dichloromethane (10 mL) was added. This mixture was subsequently stirred at room temperature under nitrogen for 2.5 h. The solution was then washed with water (25 mL), which was extracted with dichloromethane, dried with Na_2SO_4, filtered, and concentrated to a crude residue that was purified by flash column chromatography (dichloromethane/ethyl acetate 30:1). Yielded 653 mg (49%) as a white solid. R_f = 0.23 (dichloromethane/ethyl acetate 30:1). MALDI-TOF MS: Calculated mass of $C_{34}H_{57}NO_4S_2$ = 607.37. Observed $[M+Na]^+$ as m/z = 630.33. ^1H NMR (400 MHz, Chloroform-d) δ 6.68 (dd, J = 16.6, 9.8 Hz, 1H), 6.48 (d, J = 16.6 Hz, 1H), 6.22 (d, J = 9.8 Hz, 1H), 5.38 (dt, J = 4.7, 2.0 Hz, 1H), 4.94 (s, 1H), 4.49 (dt, J = 11.5, 6.3 Hz, 1H), 3.36 (q, J = 6.2 Hz, 2H), 3.29 - 3.19 (m, 2H), 2.95 - 2.85 (m, 2H), 2.70 (t, J = 6.6 Hz, 2H), 2.39 - 2.22 (m, 2H), 2.05 - 1.76 (m, 5H), 1.65 - 0.80 (m, 33H), 0.67 (s, 3H). ^{13}C NMR (101 MHz, $CDCl_3$) δ 156.2, 139.9, 136.2, 131.5, 122.8, 74.8, 56.8, 56.3, 54.5, 50.2, 42.5, 40.0, 39.9, 39.7, 38.7, 37.1, 36.7, 36.3, 35.9, 32.6, 32.1, 32.0, 28.4, 28.3, 28.2, 24.4, 24.1, 24.0, 23.0, 22.7, 21.2, 19.5, 18.9, 12.0.

(d) *MK084*: A solution of the peptide "CSIINFEKL" (47.5 mg, 0.045 mmol) in NMP (4.4 mL) was added a solution of *Cholesterol-VS* (32.5 mg, 0.053 mmol) in NMP (0.3 mL). The mixture was stirred at 75°C under nitrogen for 8 h. The crude product was purified by semi-prep HPLC. Yielded 32.2 mg (43%). HPLC (>95%). MALDI-TOF MS: Calculated mass for $C_{82}H_{136}N_{12}O_{18}S_3$ = 1672.93. Observed $[M+Na]^+$ as m/z = 1695.97

Supplementary Figures

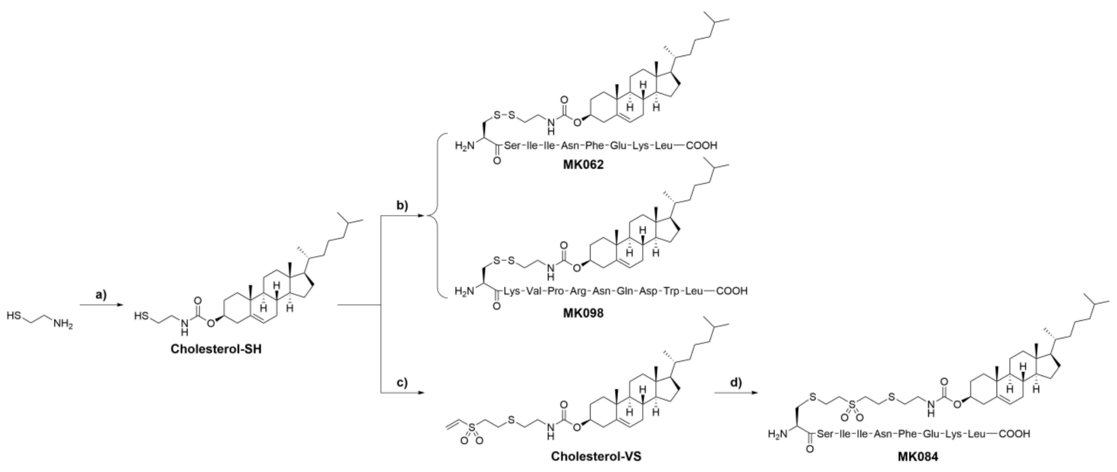

Figure S1: Synthesis of peptide antigens MK062, MK084, and MK098. *Reagents and conditions*: **(a)** Cholesteryl chloroformate, tetrahydrofuran, 0°C → reflux, 30 min; **(b)** N-terminal cysteine modified peptide, 4,4′-dipyridyl disulfide, N-methyl-2-pyrrolidinone (NMP), 4°C, 5 h; **(c)** divinyl sulfone, triethylamine, dichloromethane, room temperature, 2.5 h; **(d)** N-terminal cysteine modified peptide, NMP, 75°C, 8 h.

Formulation	*Size (nm)*	*PDI (au)*	*Z-pot (mV)*	*Lipid (mM)*	*Antigen (mM)*	*TMX-201*
MK062:TMX	114.0 ± 1.4	0.1	-15.0 ± 0.3	26.3	0.6	0.8
MK098:TMX	123.6 ± 1.4	0.1	-17.6 ± 0.3	31.2	0.5	0.9
MK062	128.2 ± 0.3	0.0	-14.9 ± 0.3	17.4	0.2	N/A
TMX-201	117.1 ± 0.5	0.0	-15.1 ± 0.7	33.2	N/A	1.0
MK084:TMX	112.0 ± 0.7	0.1	-19.8 ± 0.6	26.1	0.4	0.9

Figure S2: Liposome characteristics. Values are reported as the mean ± standard deviation

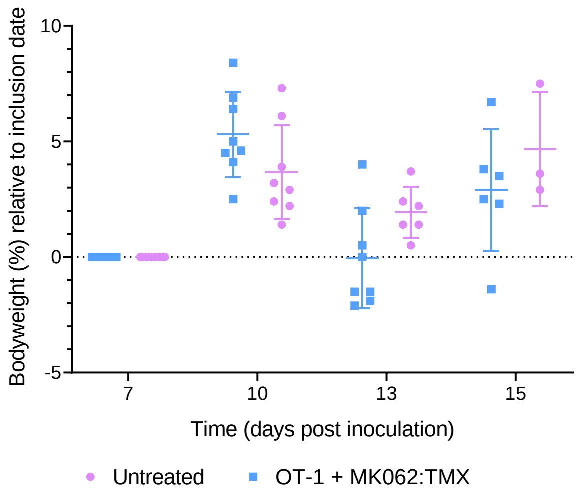

Figure S3. The combination of ACT and antigen delivery is well tolerated in mice. Female C57bl/6 mice were treated with MK062:TMX liposomal corresponding to a dose of 10 µg SIINFEKL peptide. Changes in bodyweight were measured alongside changes in tumor volume, 3 times weekly. The graph shows one representative study, with n = 8. Each symbol represents one mouse and error bars represent the mean ± SD.

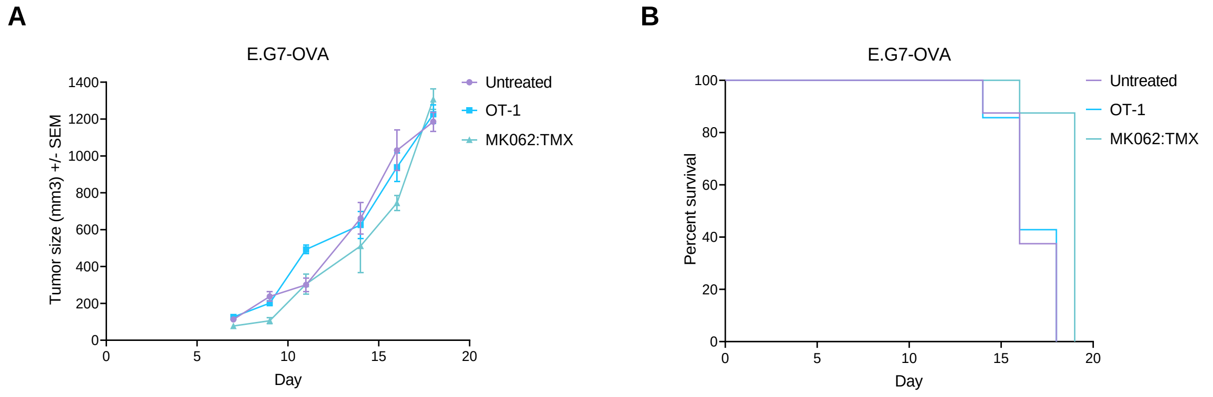

Figure S4: Transfer of previously unstimulated T cells or liposomal antigen delivery have no therapeutic effect when administered as monotherapies. (**A**) growth curves (**B**) and survival curves of mice receiving either OT-1 splenocytes or liposomes as indicated. Tumor growth was monitored by a blinded measurer, and mice were euthanized when their tumors exceeded 1000 mm3. Graphs represent pooled data from two independent experiments with n=8 mice/group in each experiment.

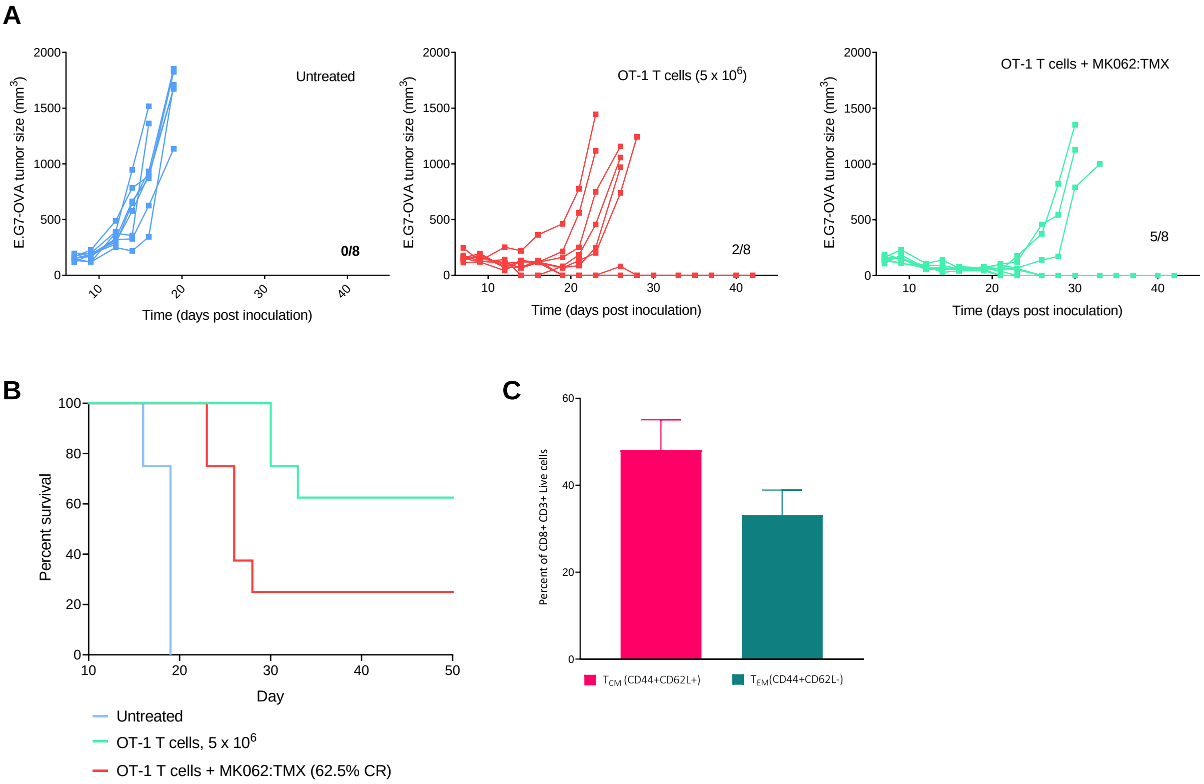

Figure S5: Liposomal antigen delivery potentiates the therapeutic efficacy of adoptively transferred memory T cells. (**A**) Individual growth curves (**B**) and survival curves of mice receiving either no treatment, expanded OT-1 CD8+ T cells or T cells and liposomes as indicated. Tumor growth was monitored by a blinded measurer, and mice were euthanized when their tumors exceeded 1000 mm^3. Graphs represent pooled data from one representative experiment with n=8 mice/group. **(C)** The phenotype of T cells analyzed immediately prior to infusion using flow cytometry. The graph shows data from 2 biological replicates within the same efficacy study. Error bars represent the mean and SD.

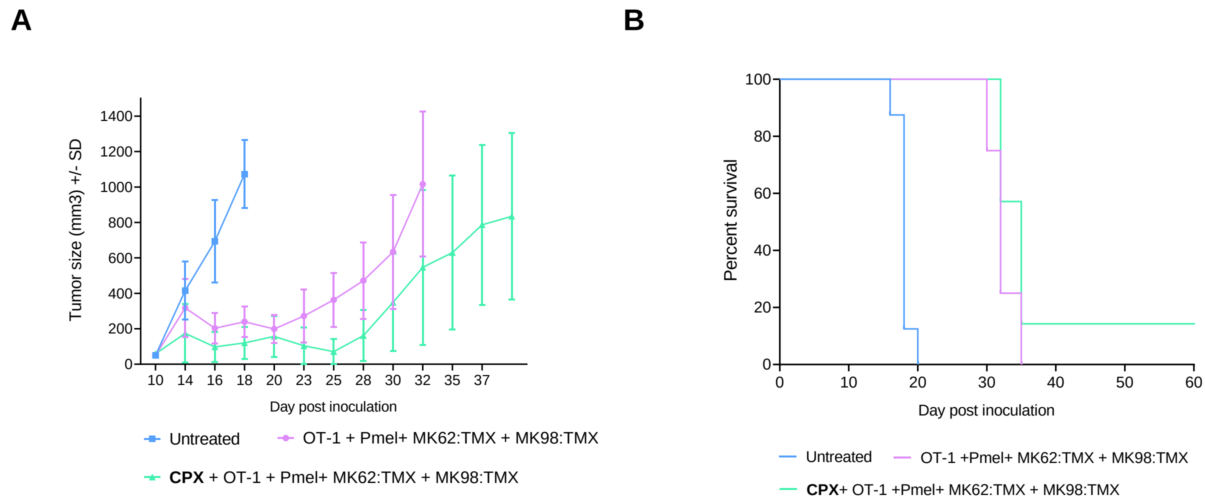

Figure S6: Addition of lymphodepleting chemotherapy prior to ACT and liposomal antigen delivery does not increase therapeutic effect. (A) Growth curves and **(B)** survival curves from one experiment in the B16-OVA model with n=8. Preconditioning was done as CPX injections, 200 mg/kg one day prior to ACT with a combination of pmel and OT-1 splenocytes, and subsequent liposomal antigen delivery with cognate peptide antigens.

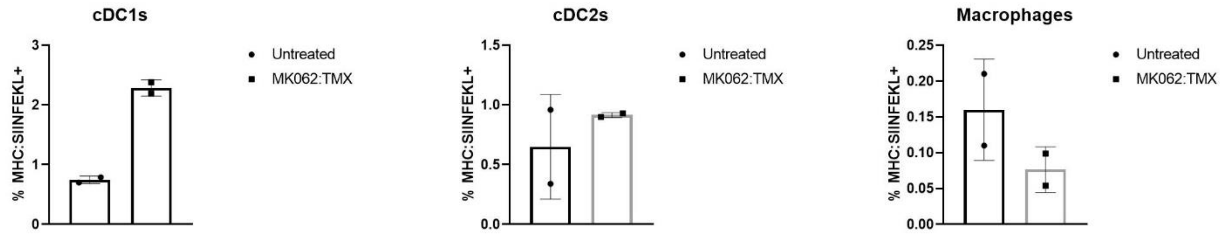

Figure S7: Antigen-presentation by splenic professional APCs. SIINFEKL antigen presentation by splenic cDC1s, cDC2s and macrophages in response to MK062:TMX treatment. C57bl/6 mice were treated with MK062:TMX liposomes corresponding to a dose of 10 µg SIINFEKL peptide or left untreated. 24 hours after treatment, antigen presentation was evaluated using an antibody specific for SIINFEKL bound to H-2Kb (MHC class I). Results are depicted as the percentage of MHC:SIINFEKL$^+$ cells out of the total population of cDC1s, cDC2s or macrophages. cDC1s were

classified as viability dye (VD)$^-$, CD45$^+$, CD64$^-$, CD26+, CD11c+, MHC II+ and CD8a+. cDC2s were classified as viability dye (VD)$^-$, CD45$^+$, CD64$^-$, CD26+, CD11c+, MHC II+ and CD11b+. Macrophages were classified as viability dye (VD)$^-$, CD45$^+$, CD64$^+$, CD26$^-$ and CD11b$^+$.

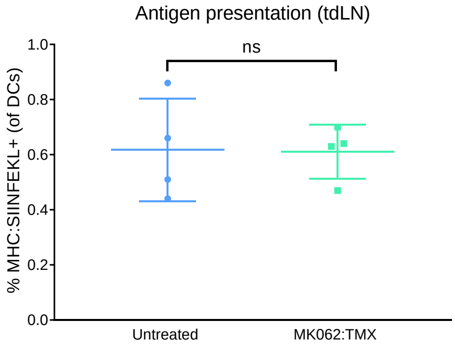

Figure S8: Antigen-presentation by DCs in the tumor-draining lymph nodes (tdLN) is not enhanced by MK062:TMX liposome treatment. Female C57bl/6 mice were treated with MK062:TMX liposomal corresponding to a dose of 10 µg SIINFEKL peptide. tdLN were excised from the treated and untreated control mice 24 hours after treatment and stained for flow cytometry analysis for evaluation of antigen presentation by DCs. Data was analyzed using FlowJo version 10. Statistics (t-test) were done using Graph Pad Prism (8.1.1) software.

A

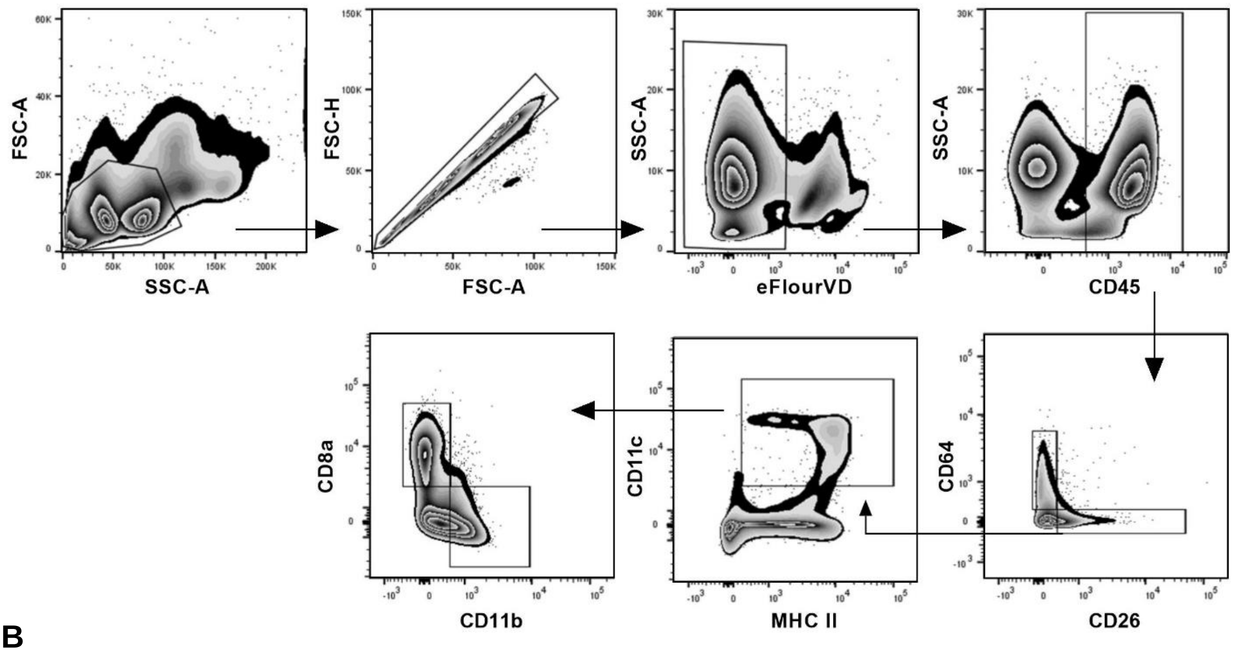

B

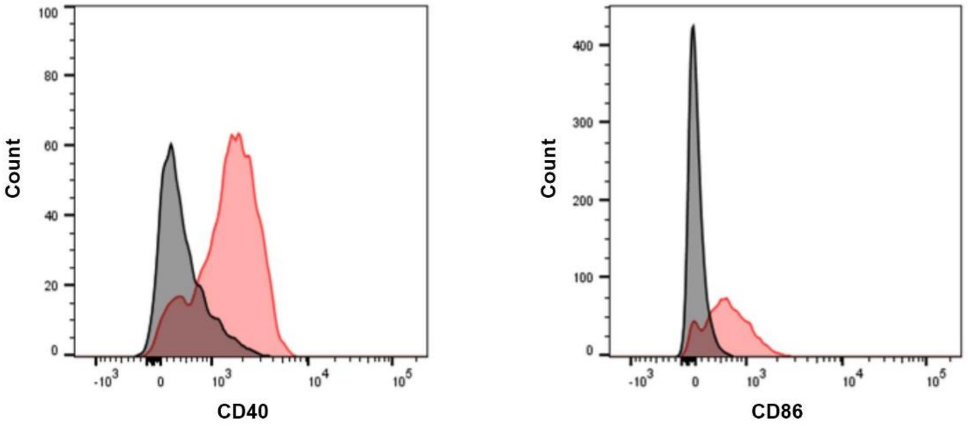

Figure S9: cDC1 activation *in vivo*. (A) Gating strategy for the identification of cDC1s that were defined as viability dye (VD)$^{-}$, CD45^{+}, CD64^{-}, CD26+, CD11c+, MHC II+ and CD8+. (**B**) Histogram showing the shift in CD40 or CD86 fluorescence intensity by splenic cDC1s in MK062:TMX treated mice (red) compared to splenic cDC1s from untreated control mice (black). Female C57bl/6 mice were treated with MK062:TMX liposomes corresponding to a dose of 10 µg SIINFEKL peptide and

10 µg TMX-201. Spleens were excised from the treated and untreated control mice 24 hours after treatment and stained for flow cytometry analysis for evaluation of expression levels of CD40 and CD86 by splenic cDC1s.

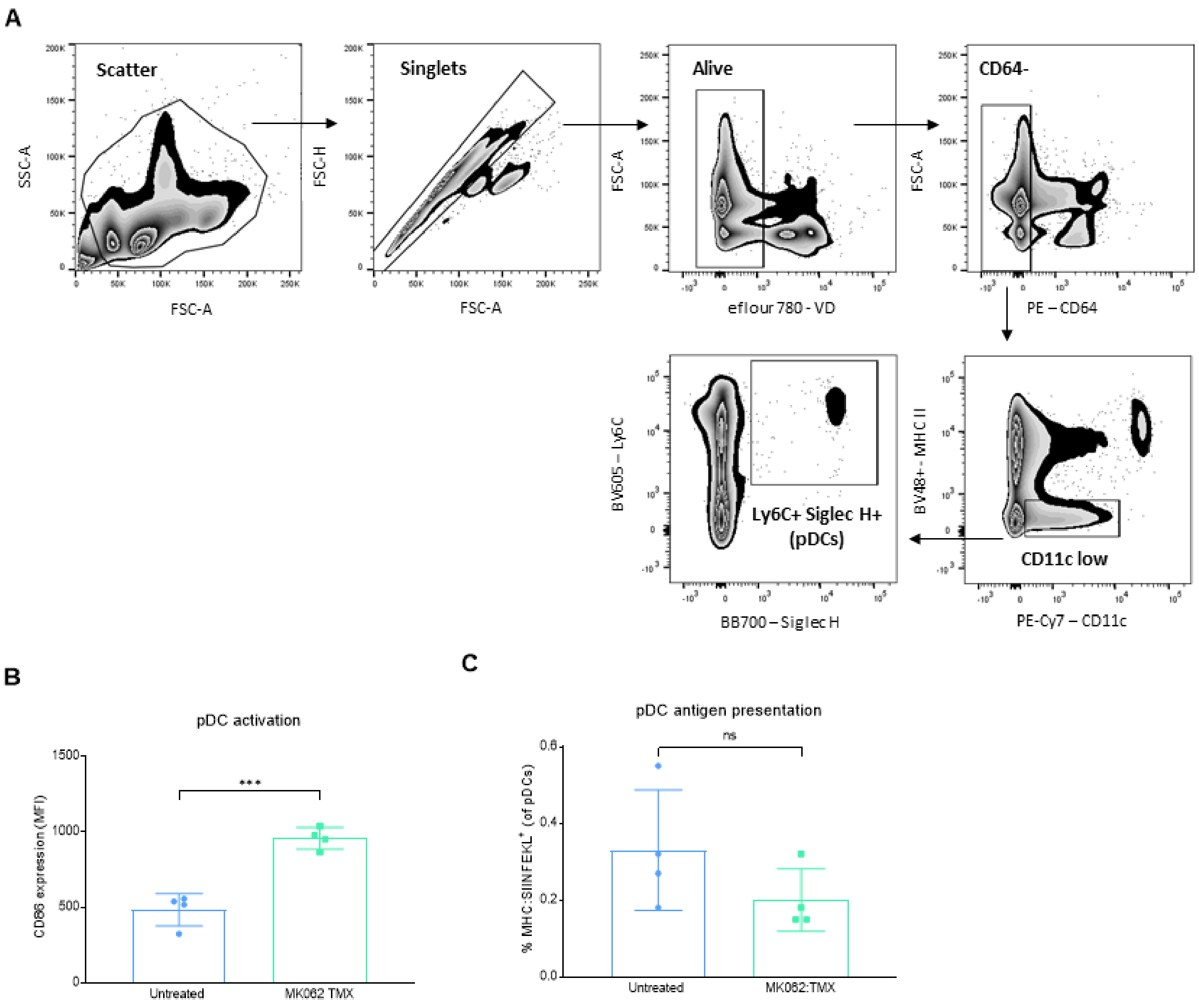

Figure S10: Splenic pDCs are activated but do not present antigen upon liposomal treatment. (A) Exemplified splenic pDC gating strategy. Spleens from female C57bl/6 were excised and stained for flow cytometry analysis. pDCs were identified as viability dye (VD)$^{-}$, CD45^{+}, CD64^{-}, CD11c^{lo}, Siglec-H^{+} and Ly6C^{+}. Data was analyzed using FlowJo version 10.

(B) Activation and **(C)** SIINFEKL antigen presentation of splenic pDCs following treatment with MK062:TMX liposomes. Female C57bl/6 mice were treated with MK062:TMX liposomes corresponding to a dose of 10 µg SIINFEKL peptide. Spleens were excised from the treated and untreated control mice 24 hours after treatment and stained for flow cytometry analysis for evaluation of activation and antigen presentation by pDCs. Data was analyzed using FlowJo version 10. Statistics (t-test) were done using Graph Pad Prism (8.1.1) software. ***P ≤ 0.001.

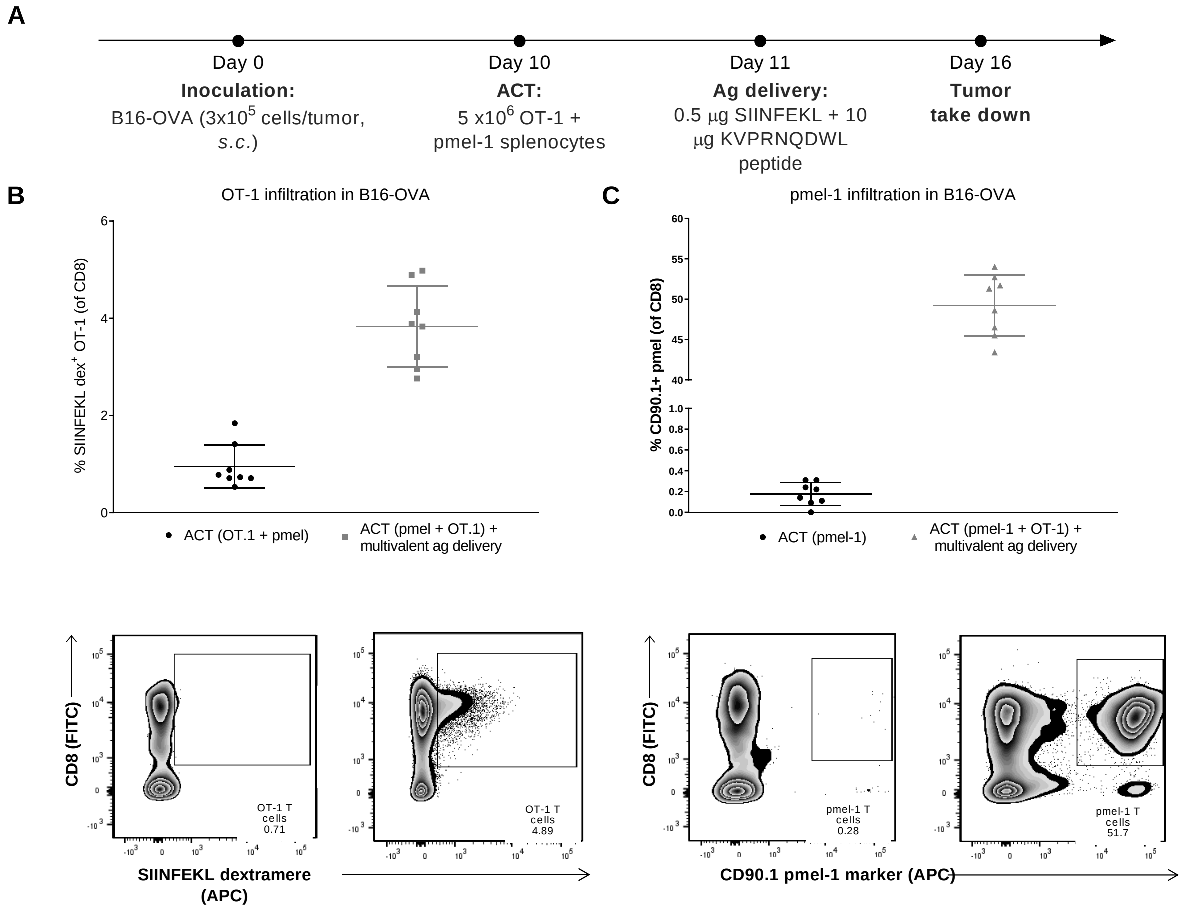

Supplementary figure S11: ACT and dual-valent antigen delivery expands multiple T cell subsets simultaneously in the tumor

A: Dosing schedule for flow cytometric analysis of T cell infiltration. Mice bearing palpable tumors were treated with ACT on day 10 as monotherapy or in combination with multivalent liposomal

antigen delivery on day 11. Five days later (on day 16), tumors were harvested for flow cytometry. **B:** Percentage of OT-1 CD8$^+$ T cells in B16-OVA tumors. **C:** Percentage of pmel-1 CD8$^+$ T cells in B16-OVA tumors. Representative flow plots are shown below each graph.

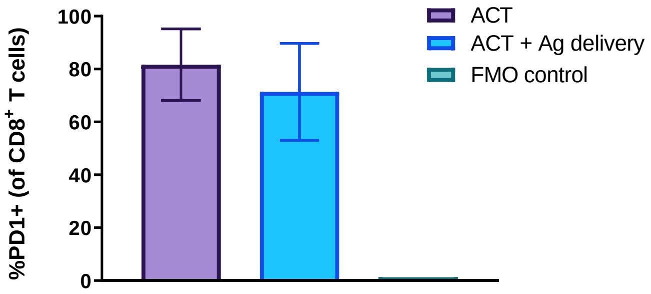

Supplementary figure S12: Intratumoral CD8$^+$ T cells show high levels of PD-1 expression. To evaluate PD-1 expression of CD8$^+$ T cells in the tumor, a mechanistic study was carried out in EG.7-OVA. C57bl/6 female mice bearing established EG.7-OVA tumors were treated with OT-1 splenocytes (ACT, n=4), or OT-1 splenocytes and liposomes carrying peptide and adjuvant (ACT + Ag delivery, n=4). For analysis, tumors were harvested and analyzed for expression of PD-1 using flow cytometry. A fluorophore minus one (FMO) control for PD-1 antibody staining is also shown (n=1).

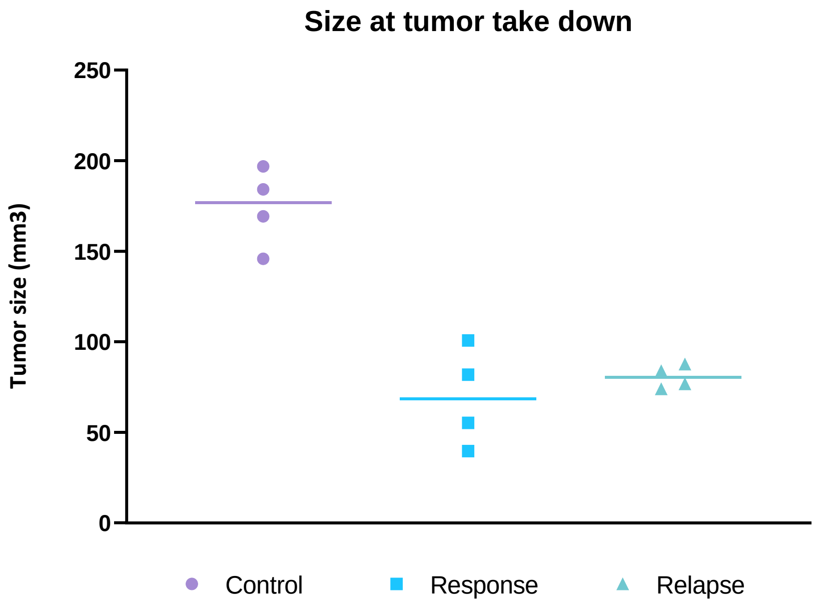

Supplementary figure S13: Tumor sizes at takedown for RNA isolation and sequencing. To minimize bias related to tumor size, we aimed for a takedown when the tumor size reached approximately 100 mm^3. C57bl/6 female mice bearing established EG.7-OVA tumors were either left untreated, treated with *iv.* injection of OT-1 splenocytes (ACT) or a combination of OT-1 splenocytes and MK062:TMX liposomes. Tumor size was measured 3-5 times weekly. The graph shows tumor sizes of individual mice at takedown.

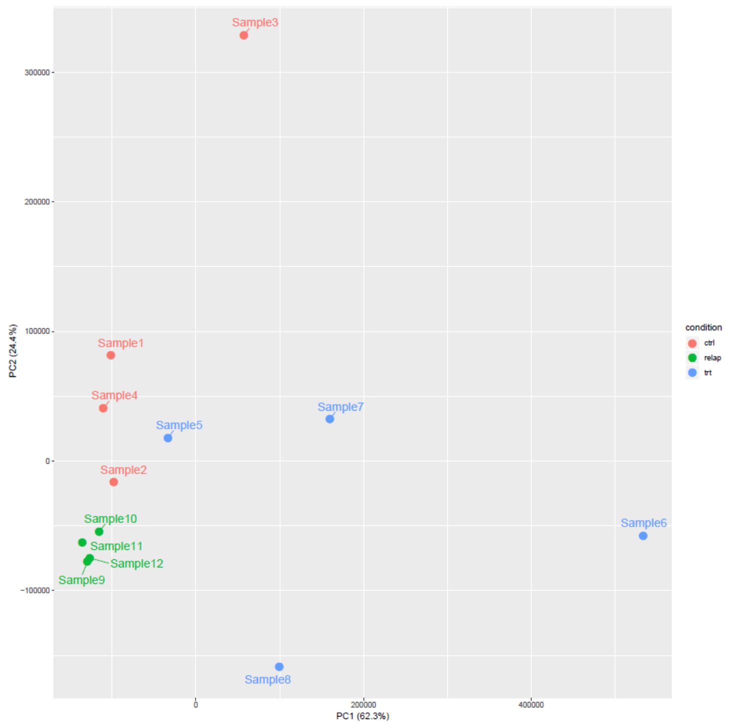

Supplementary figure S14: Principle component analysis.

Principle component analysis plot of the 12 RNA-seq samples colored by group. Red: Control (ctrl), green: relapse (relap), blue: treatment (trt).
